# Plasma tau biomarkers for biological staging of Alzheimer’s disease

**DOI:** 10.1038/s43587-025-00951-w

**Published:** 2025-08-22

**Authors:** Laia Montoliu-Gaya, Gemma Salvadó, Joseph Therriault, Johanna Nilsson, Shorena Janelidze, Sophia Weiner, Nicholas J. Ashton, Andrea L. Benedet, Nesrine Rahmouni, Juan Lantero-Rodriguez, Niklas Mattsson-Carlgren, Sebastian Palmqvist, Gunnar Brinkmalm, Erik Stomrud, Henrik Zetterberg, Johan Gobom, Pedro Rosa-Neto, Kaj Blennow, Oskar Hansson

**Affiliations:** 1 Department of Psychiatry and Neurochemistry, Institute of Neuroscience & Physiology, The Sahlgrenska Academy at the University of Gothenburg, Mölndal, Sweden; 2 Clinical Memory Research Unit, Department of Clinical Sciences Malmö, Lund University, Lund, Sweden; 3 Translational Neuroimaging Laboratory, McGill Research Centre for Studies in Aging, McGill University, Montreal, Quebec Canada; 4 Department of Neurology and Neurosurgery, Faculty of Medicine, McGill University, Montreal, Quebec Canada; 5 Banner Sun Health Research Institute, Sun City, AZ USA; 6 Banner Alzheimer’s Institute, Phoenix, AZ USA; 7 Wallenberg Center for Molecular Medicine, Lund University, Lund, Sweden; 8 Memory Clinic, Skåne University Hospital, Malmö, Sweden; 9 Clinical Neurochemistry Laboratory, Sahlgrenska University Hospital, Mölndal, Sweden; 10 Department of Neurodegenerative Disease, Queen Square Institute of Neurology, University College London, London, UK; 11 UK Dementia Research Institute, University College London, London, UK; 12 Hong Kong Center for Neurodegenerative Diseases, Hong Kong, China; 13 UW Department of Medicine, School of Medicine and Public Health, Madison, WI USA; 14 Paris Brain Institute, ICM, Pitié-Salpêtrière Hospital, Sorbonne University, Paris, France; 15 Neurodegenerative Disorder Research Center, Division of Life Sciences and Medicine, and Department of Neurology, Institute on Aging and Brain Disorders, University of Science and Technology of China and First Affiliated Hospital of USTC, Hefei, China

**Keywords:** Diagnostic markers, Alzheimer's disease

## Abstract

A blood biomarker-based staging system for Alzheimer’s disease (AD) could improve the diagnosis, prognosis and identification of individuals most likely to benefit from specific therapies. Here, using targeted mass spectrometry, we measured six phosphorylated and six nonphosphorylated tau peptides in plasma from two independent cohorts: BioFINDER-2 and TRIAD (*n* = 689). We also analyzed the ratios of phosphorylated to nonphosphorylated peptides. Our results revealed that specific tau species became abnormal at different points along the disease continuum. Based on these findings, we developed a data-driven, blood-based staging model that demonstrated strong consistency across cohorts (>85% agreement in ≥90% initializations) and reflected changes in other AD biomarkers. These plasma-based stages were associated with clinical diagnoses, positron emission tomography-based stages and distinct patterns of longitudinal disease progression, including Aβ- and tau-positron emission tomography uptake, atrophy and cognitive decline. This study highlights the potential of tau blood-based biomarkers for biological staging in AD, offering a scalable tool for tracking disease progression and guiding clinical decisions.

## Abstract (web summary)

Montoliu-Gaya, Salvadó et al. develop a blood-based model using tau biomarkers measured in a single analysis enabling biological staging of Alzheimer’s disease to support the diagnosis, prognosis and identification of patients likely to benefit from targeted therapies.

## Main

Alzheimer’s disease (AD) is the leading cause of dementia worldwide, affecting more than 55 million patients^1^. The recent approval of amyloid-β (Aβ)-targeting immunotherapies for the treatment of mild-to-moderate AD underscores the need for accessible biomarkers, not only for detecting the disease pathologies but also staging such pathologies for optimal treatment initiation and evaluating outcomes^2^. Staging systems that model disease progression can provide a framework for detecting and monitoring pathological changes over its long course and assist in clinical decision-making^3^. For example, evidence indicates that patients with AD who exhibit lower tau tracer uptake on positron emission tomography (PET) scans respond better to Aβ-targeting monoclonal antibody therapies, suggesting that this group may have the most favorable risk–benefit ratio for this treatment^4,5^. It is likely that other therapies, such as tau-targeting therapies, will also prove to be more beneficial at certain biological disease stages. However, PET scans are costly and require specialized personnel and facilities^6,7^. Implementing a panel of fluid biomarkers, especially in blood, would substantially enhance the accessibility and cost-effectiveness of biomarker-based AD staging.

Although fluid biomarkers do not provide brain topographical information on Aβ and tau pathology, as imaging biomarkers do^8^, emerging evidence suggests that various tau species could be used for staging the disease. In the AD brain, pathological hyperphosphorylation of tau occurs sequentially at different sites along the sequence of the protein^9,10^. As the disease advances, both the number of phosphorylation sites and the extent of their occupancy increase, aligning with the gradual rise in the molecular weight of tau aggregates and overall disease progression^9^. This differential pattern of tau phosphorylation has been observed in cerebrospinal fluid (CSF), where site-specific phosphorylation changes follow distinct trajectories over time^11,12^. For instance, phosphorylations such as p-tau181, p-tau217 and p-tau231 begin to rise in CSF concurrently with the early increase in Aβ aggregates^11,13^, while tau phosphorylation at position 205 may rise later and be more strongly associated with tau-PET measures^14,15^. Nonphosphorylated tau species, such as N-terminal tau fragments (NTA)^16,17^, total tau (T-tau) measured with mid-domain sandwich assays^18^, MTBR-tau243 (refs. ^19,20^) or nonphosphorylated tau peptides^11,21^, have been shown to increase in CSF at later stages, with even stronger associations with tau-PET and cognition. The distinct emergence of phosphorylated and nonphosphorylated tau biomarkers has been leveraged to create a CSF staging model^22^, where more advanced stages correlate with higher amyloid and tau-PET uptake and greater cognitive impairment. These CSF stages also predict the longitudinal trajectories of imaging biomarkers and clinical progression. Yet, the potential use of tau blood-based biomarkers for staging AD remains in its early stages. Multiple studies using different assays and platforms suggest that the pattern observed in CSF might also be detectable in blood^23–26^. The Alzheimer’s Association (AA) recently published revised criteria for the diagnosis of AD^27,28^, updating the previous guidelines published in 2018^29^. These new criteria also include a biomarker framework for staging AD using amyloid and tau-PET imaging biomarkers, as well as a conceptual biological staging model based on fluid biomarkers. However, further investigation and validation are necessary to confirm the utility of fluid tau biomarkers, particularly those in blood, for staging AD—an advancement that would be highly valuable for patient management.

In this study, we examined how various plasma tau biomarkers become abnormal across the AD continuum and evaluated their ability to stage the disease. We utilized a targeted mass spectrometry (MS) method that systematically quantifies multiple phosphorylated and nonphosphorylated plasma tau species in a single analysis^30,31^. Specifically, we measured levels of six phosphorylated and six nonphosphorylated tau peptides in plasma samples from two independent cohorts of sporadic AD: the Swedish BioFINDER-2 study (*n* = 549) and Translational Biomarkers in Aging and Dementia (TRIAD) cohort (*n* = 140). We first assessed the point at which the levels of different plasma tau biomarkers became abnormal over the course of the clinical disease stages in the BioFINDER-2 cohort and developed a staging model based on selected biomarkers. This model was subsequently applied to the BioFINDER-2 cohort and independently validated in the TRIAD cohort. We then compared the distribution of imaging biomarkers and cognitive measures across the plasma stages, and explored associations between plasma stages, clinical diagnosis and imaging stages in accordance with the new AA criteria^27,28^. Finally, using longitudinal data in the BioFINDER-2 cohort, we determined the rate and trajectory of Aβ and tau-PET accumulation, cognitive decline and neurodegeneration for individuals at each plasma stage.

## Results

### Participant characteristics and plasma tau biomarker measures

The BioFINDER-2 cohort included 549 individuals: 191 were cognitively unimpaired Aβ negative (CU−), 82 were cognitively unimpaired Aβ positive (CU+), 76 were mildly cognitively impaired and Aβ positive (MCI+) and 80 had AD dementia and were Aβ positive (ADdem+). A subset of participants exhibited other neurodegenerative diseases than AD (95 Aβ negative and 18 Aβ positive). The average age was 70.4 ± 12.5 years (mean ± s.d.), 284 (51.7%) were women and 281 (51.3%) were *APOE* ε4 carriers. The TRIAD cohort included a total of 140 participants, distributed in groups across the AD continuum: 34 CU−, 25 CU+, 35 MCI+ and 28 AD dementia, while 7 exhibited other neurodegenerative diseases. The average age was 71.5 ± 6.5 years, 58 (41.4%) were women and 61 (43.6%) were *APOE* ε4 carriers. Demographic information is presented in Table 1. In BioFINDER-2, a subset of participants had available longitudinal data on Aβ-PET (*n* = 368), tau-PET (*n* = 480) and cortical thickness (*n* = 457).

**Table 1:** Participant characteristics in the BioFINDER-2 and TRIAD cohorts

	BioFINDER-2 (*n* = 549)	TRIAD (*n* = 140)	*P* value
Age, years	70.4 (12.5)	71.5 (6.5)	0.140
Women, *n* (%)	284 (51.7%)	58 (41.4%)	0.176
Education years	12.4 (3.7) [*n* = 545]	15.6 (3.49)	<0.001
*APOE* ε4 carriership, *n* (%)	281 (51.3%) [*n* = 548]	61 (43.6%)	0.125
Diagnosis, *n* (%)			<0.001
CU−	191 (34.8%)	34 (24.3%)	
CU+	82 (14.9%)	25 (17.9%)	
MCI+	76 (13.8%)	35 (25.0%)	
AD dementia	80 (14.6%)	28 (20.0%)	
Cognitively impaired not due to AD	113 (20.6%)	7 (5.0%)	
Other	7 (1.3%)	11 (7.9%)	
Aβ positivity, *n* (%)	256 (47.2%) [*n* = 542]	88 (62.9%) [*n* = 129]	<0.001
Tau-PET positivity, *n* (%)	150 (27.5%) [*n* = 545]	54 (38.6%) [*n* = 127]	0.001

Mean (s.d.) shown unless otherwise specified. In characteristics in which some data were missing, we include the final data sample in square brackets.

Plasma tau biomarkers were measured in the BioFINDER-2 and TRIAD cohorts using a targeted MS method^30^. This approach quantifies the levels of six phosphorylated tau species (p-tau181, p-tau199, p-tau202, p-tau205, p-tau217 and p-tau231) and six nonphosphorylated tau peptides (tau195-209, tau212-221, 0N-tau, 1N-tau, PNS131-138 and PNS275-291). The nonphosphorylated tau species 195-209 and 212-221 are common to all tau isoforms, whereas the 0N and 1N-tau peptides are specific to the 0N and 1N tau isoforms, respectively. The PNS peptides are unique to the PNS-tau isoform, which is primarily expressed in the peripheral nervous system (PNS)^32^.

### Plasma tau biomarkers emerge differentially across the AD continuum

**Fig. 1:**
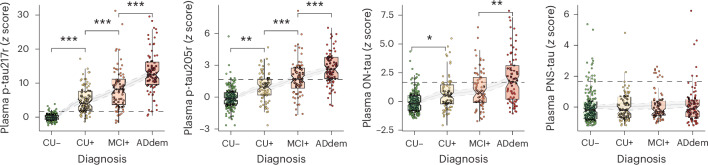
Plasma tau biomarker levels by diagnosis and Aβ status. Levels were *z*-scored using the CU− group as a reference. The horizontal dashed line is set at *z* = 1.96, which represents the abnormality (95% CI of the CU− group). Individuals with non-AD diagnosis were not included in this analysis. Plasma p-tau217r and p-tau205r are ratio metrics calculated by dividing the intensity of the phosphorylated peptide by that of its corresponding nonphosphorylated peptide. The represented PNS peptide data correspond to PNS275-291. Statistical differences by diagnostic groups were assessed using ANCOVA adjusted by age and sex and *APOE* ε4 carriership followed by Tukey’s corrected, post-hoc pairwise comparisons (two-sided analysis). In the figure, only differences between contiguous groups are shown. Boxplots summarize the data distribution, showing the median (central line), interquartile range (IQR; box) and whiskers extending to 1.5× the IQR. All individuals with available data were included (*n* = 549). Exact *P* values can be found in Supplementary Table 1. *P < 0.05, **P < 0.005, ***P < 0.001. CU−, cognitively unimpaired Aβ-negative; CU + , cognitively unimpaired Aβ-positive; FDR, false discovery rate; MCI + , mild cognitive impairment Aβ-positive; ADdem, Alzheimer’s disease dementia.

**Extended Data Fig. 1:**
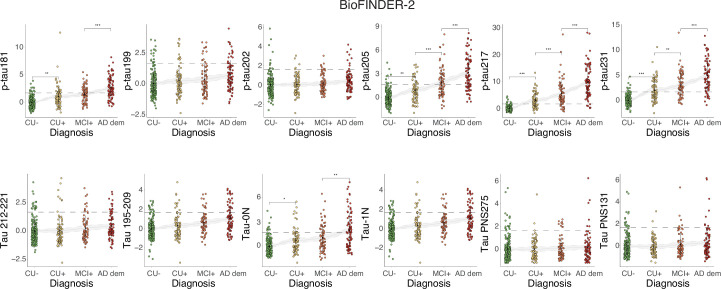
Plasma tau biomarkers levels by diagnostic and Aβ status. Levels were z-scored using CU- group as a reference. Horizontal dashed line is set at z = 1.96, which represents the abnormality (95%CI of CU- group). Individuals with non-AD diagnosis were not included in this analysis. Statistical differences by diagnostic groups were assessed using ANCOVA adjusted by age and sex and *APOE ε*4 carriership followed by Tukey’s corrected, post hoc pairwise comparisons (two-sided analysis). In the figure, differences only differences by contiguous groups are shown. Mean (black dot) and SD (vertical black line) plasma levels by diagnostic group are shown. Exact p-values can be found in Supplementary Table 1.*: p < 0.05; **: p < 0.005; ***: p < 0.001. Abbreviations: CU-, cognitively unimpaired Aβ-negative; CU + , cognitively unimpaired Aβ-positive; FDR, false discovery rate; MCI + , mild cognitive impairment Aβ-positive; ADdem, Alzheimer’s disease dementia.

We first examined how the levels of plasma tau biomarkers varied across diagnostic groups based on cognitive status and Aβ status (Fig. 1, Extended Data Fig. 1 and Supplementary Table 1). Apart from the quantified peptides, we additionally included in the analysis the ratios between the phosphorylated peptides p-tau217 and p-tau205 and their respective nonphosphorylated forms: p-tau217/212-221 (p-tau217r) and p-tau205/195-209 (p-tau205r). Using a threshold based on the mean plus 1.96 standard deviations of the CU− group (which corresponds to its 95% confidence interval (CI)) to define biomarker abnormality, we found that the different tau species showed significant changes to positivity at distinct points across the continuum. Plasma p-tau217 (*P* < 0.001), p-tau217r (*P* < 0.001) and p-tau231 (*P* < 0.001) exhibited abnormal levels in the CU+ stage. Plasma p-tau205 (*P* < 0.001) and p-tau205r (*P* < 0.001) values became abnormal at the MCI+ stage, while p-tau181 (*P* < 0.001) and 0N-tau (*P* = 0.001) levels changed to positivity in the ADdem+ group. Other p-tau biomarkers, including p-tau199, p-tau202 and the nonphosphorylated species tau195-209, tau212-221 and 1N-tau, presented some significant increases among diagnostic groups, but never above the positivity threshold. The peripheral-specific (PNS131-138 and PNS275-291) tau peptides did not show any significant change in their levels with disease progression.

### Plasma tau biomarker staging model

Based on these findings, we developed a plasma staging model using biomarkers with distinct abnormal changes along the continuum. Among biomarkers with changes to abnormality occurring at the same clinical stage, p-tau217r was chosen over p-tau231 and 0N-tau over p-tau181, due to their greater significant changes across the disease continuum (Supplementary Table 1). In addition, the ratios p-tau217r and p-tau205r were selected over the phosphorylated tau species alone because ratios of p-tau to nonphosphorylated tau may help minimize confounding effects from medical comorbidities such as kidney disease. To explore this, we performed a sensitivity analysis stratifying participants by chronic kidney dysfunction status based on estimated glomerular filtration rate measures (Supplementary Table 2). We found that all tau biomarkers, including PNS-tau, were significantly altered in individuals with kidney dysfunction, but this effect was mitigated when using the phospho/nonphospho ratios, corroborating previous findings^33^.

**Extended Data Fig. 2:**
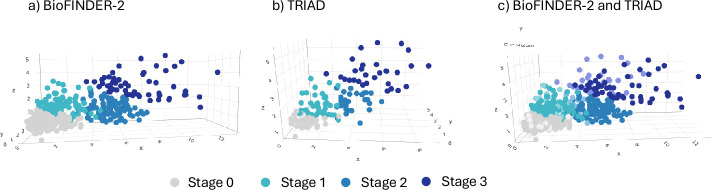
Clustering classification of plasma stages. 3D-plot including the three biomarkers used for the clustering approach (x-axis: p-tau217r, y-axis: p-tau205r and z-axis: Tau-0N) for BioFINDER-2 (**a**), TRIAD (**b**), and both BioFINDER-2 and TRIAD (**c**). Colors represent the plasma stages as classified by the clustering approach. In **c**, TRIAD individuals are shown in the same color but with lighter shade in comparison to BioFINDER-2 individuals.

**Extended Data Fig. 3:**
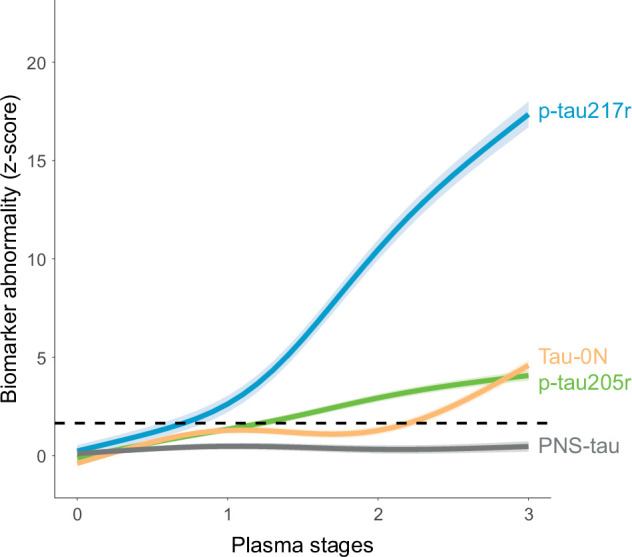
Selected tau biomarker trajectories by plasma stages in the BioFINDER-2 cohort. LOESS regression of the plasma biomarkers included in the staging model plus plasma PNS-tau for comparison. Biomarker levels are z-scored based on a group of CU− participants, and all increases represent an increase in abnormality. Colored lines and bands represent the LOESS regression and its 95% CI. Horizontal line is drawn at z-score = 1.96, which represents 95% CI of the reference group (CU−). Abbreviation: LOESS, locally estimated scatterplot smoothing.

**Extended Data Fig. 4:**
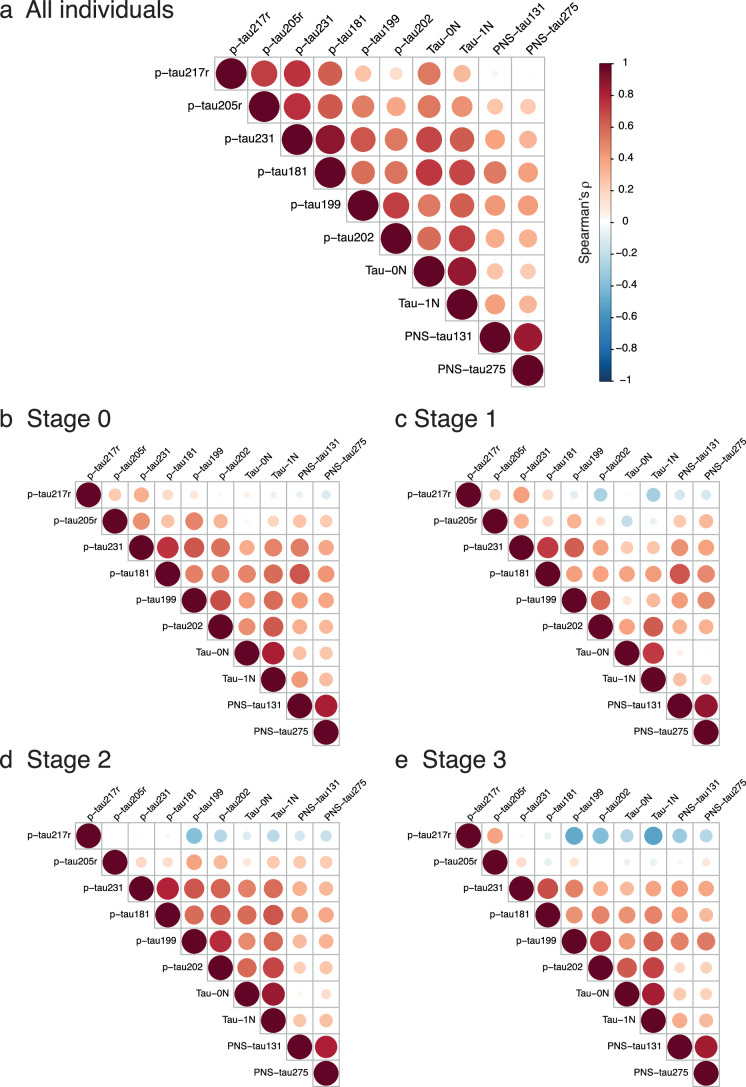
Cross-correlation among plasma biomarkers. Spearman’s rho correlations are shown among plasma biomarkers using all (**a**) or by plasma stages participants (**b-e**).

We first normalized the selected plasma biomarkers (p-tau217r, p-tau205r and 0N-tau), dividing by the median of the CU− group, in the two cohorts independently. We then performed *k*-means clustering to classify each individual into one of four blood-based stages, using these three biomarkers (Methods). This can be visualized as four distinct clusters within a three-dimensional space, where each biomarker represents one of the axes (Extended Data Fig. 2). The final model included a negative stage (that is, stage 0), which corresponds to normality in all biomarkers, and three positive stages (that is, stages 1–3). Locally estimated scatterplot smoothing (LOESS) representation of the change of the plasma biomarkers at each stage showed that p-tau217r p-tau205r and 0N-tau became abnormal consecutively and in accordance with the plasma stages (Extended Data Fig. 3) and presented differential correlations among tau markers at each plasma stage (Extended Data Fig. 4).

**Extended Data Fig. 5:**
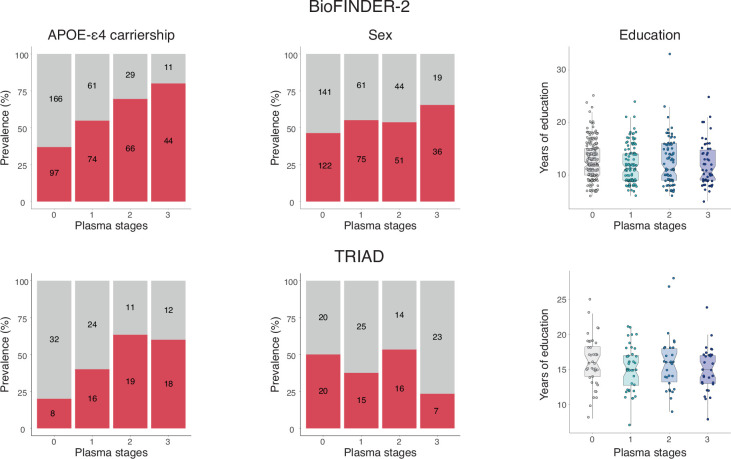
Demographic characteristics by plasma stages. For *APOE* ε4 carriership and sex, number of individuals in each group per stage are detailed in the barplots (red color represents carriers and women, respectively). Plasma stages were created in the main (BioFINDER-2) cohort and validated in the replication cohort (TRIAD). Years of education by stages defined by plasma biomarkers are also shown. Group comparisons were computed with an ANOVA, followed by Tukey-corrected *post hoc* pairwise comparisons. Only comparison between consecutive groups are shown. Boxplots summarize data distribution, showing the median (central line), interquartile range (IQR; box) and whiskers extending to 1.5 × the IQR. *: p < 0.05; **: p < 0.005; ***: p < 0.001.

The blood biomarker stages were not associated with years of education (BioFINDER-2 *P* = 0.083, TRIAD *P* = 0.210), but an increase in the proportion of *APOE* ε4 carriers was observed with advancing stages (BioFINDER-2 and TRIAD *P* < 0.001). A slightly increasing trend in the percentage of women with plasma stages was observed in BioFINDER-2 (*P* = 0.047), which was not significant in TRIAD (*P* = 0.063) (Extended Data Fig. 5).

To assess the replicability of the model, we reclassified each individual with different starting points (*n* = 5,000) and determined the percentage of occasions each individual was placed in the plasma-based same stage (Supplementary Table 3). Notably, >85% of individuals were classified in the same stage ≥90% of the occasions. In addition, we tested the model using biomarker concentrations instead of MS-calculated ratios, as they may be more applicable in clinical settings. Both models showed a relatively high correspondence (BioFINDER-2: 0.73, TRIAD: 0.69; Supplementary Table 4).

### Plasma biomarker stages show a consistent pattern in relation to imaging biomarkers and cognition

**Extended Data Fig. 6:**
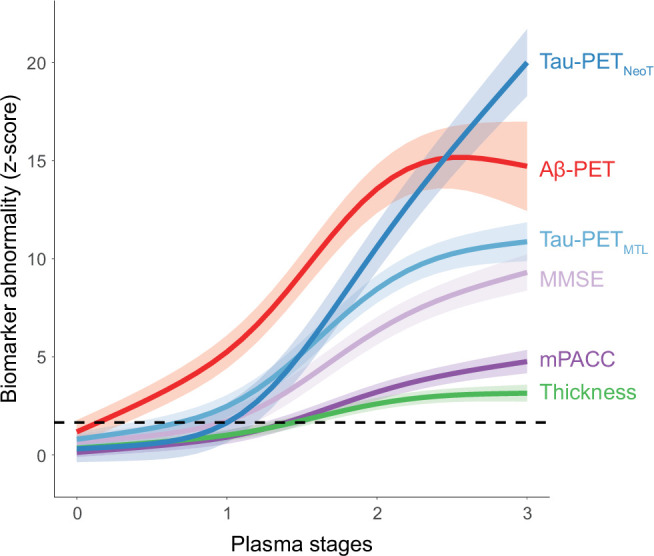
AD biomarker trajectories by plasma stages in the BioFINDER-2 cohort. LOESS regression of imaging and cognitive biomarkers. Biomarker levels are z-scored based on a group of CU− participants, and all increases represent an increase in abnormality. Colored lines and bands represent the LOESS regression and its 95% CI. Horizontal line is drawn at z-score = 1.96, which represents 95% CI of the reference group (CU−). Abbreviations: LOESS, locally estimated scatterplot smoothing. MMSE, Mini-Mental State Examination; mPACC, modified preclinical Alzheimer’s cognitive composite; MTL, medial temporal lobe; NeoT: temporal neocortex.

**Extended Data Fig. 7:**
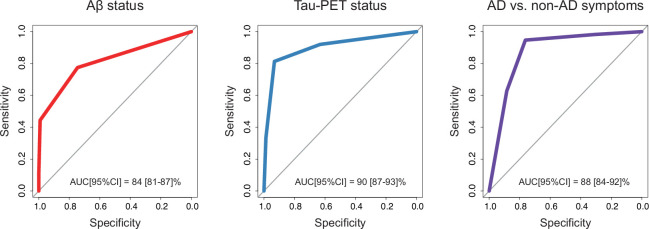
Plasma stages for predicting dichotomic categories. ROC curves were used to assess the classification of Aβ-PET, tau-PET and AD versus non-AD cognitive impairment in the BioFINDER-2 cohort. Abbreviations: AUC, area under the curve; ROC, receiver operating characteristic.

We then evaluated the relationship between the plasma stages in our model and key AD markers, including insoluble Aβ aggregates (Aβ-PET), insoluble tau aggregates (tau-PET) at mediotemporal (MTL) and temporal neocortex (NeoT) regions, neurodegeneration (cortical thickness) and cognitive performance. Regarding the plasma stages, Aβ-PET showed the earliest changes toward abnormality, followed by tau-PET MTL, tau-PET NeoT, cognitive scores and finally, cortical thickness (Extended Data Fig. 6). In addition, the model was proven accurate to predict Aβ-PET (AUC (95% CI) = 84 (81 to 87)) and tau-PET (AUC (95% CI) = 90 (87 to 93)) status as well as AD-related cognitive symptoms (AUC (95% CI) = 88 (84 to 92)) (Extended Data Fig. 7).

**Fig. 2:**
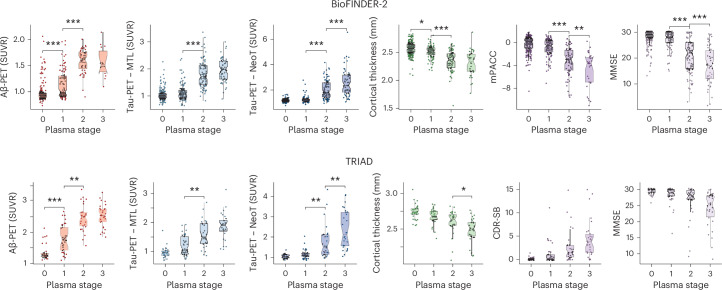
AD biomarkers by plasma stage. Biomarkers of Aβ, early (MTL) and intermediate (NeoT) tau pathology measured by PET, neurodegeneration (cortical thickness) and sensitive (mPACC or CDR-SB) and global cognition (MMSE) by stages defined by plasma biomarkers. Plasma stages were created in the main (BioFINDER-2) cohort and validated in the replication cohort (TRIAD). All individuals with available data were included in the Aβ- and tau-PET analyses, but individuals with non-AD diagnosis were excluded from cortical thickness and cognitive tests to avoid bias. Statistical differences by plasma stage were assessed using ANCOVA adjusted by age and sex and *APOE* ε4 carriership followed by Tukey’s corrected, post-hoc pairwise comparisons (two-sided analysis). In the figure, only differences between contiguous groups are shown. Boxplots summarize data distribution, showing the median (central line), interquartile range (IQR; box) and whiskers extending to 1.5× the IQR. All individuals with available data were included (Aβ-PET: *n* = 370 in BioFINDER-2 and *n* = 129 in TRIAD, MTL tau-PET and NeoT tau-PET: *n* = 476 in BioFINDER-2 and *n* = 127 in TRIAD, cortical thickness: *n* = 450 in BioFINDER-2 and *n* = 129 in TRIAD, mPACC/CDR-SB: *n* = 476 in BioFINDER-2 and *n* = 135 in TRIAD, MMSE: *n* = 529 in BioFINDER-2 and *n* = 138 in TRIAD). Exact *P* values can be found in Supplementary Table 6. *P < 0.05, **P < 0.005, ***P < 0.001.

A consistent pattern of changes in AD pathology biomarkers was observed across stages in both the BioFINDER-2 and TRIAD cohorts, supporting the validity of the model (see representation in Fig. 2, quantitative cross-stage profiles in Supplementary Table 5 and analysis of group differences in Supplementary Table 6). In the BioFINDER-2 cohort, Aβ-PET levels exhibited the first significant increase between plasma stages 0 and 1 (*β* = 0.37 (0.14 to 0.59), *P* < 0.001), followed by further elevation at stage 2 (*β* = 1.34 (1.03 to 1.65), *P* < 0.001), after which they plateaued at stage 3 (*β* = −0.04 (−0.59 to 0.5), *P* = 0.99). MTL tau-PET showed significant changes from stage 1 to stage 2 (*β* = 1.30 (1.04 to 1.56), *P* < 0.001). NeoT tau-PET exhibited significant differences between stages 1 and 2 (*β* = 1.13 (0.87 to 1.4), *P* < 0.001) and further increases between stages 2 and 3 (*β* = 0.99 (0.63 to 1.35), *P* < 0.001). Cortical thickness progressively decreased with advancing plasma stages, showing significant reductions between stages 0 and 1 (*β* = −0.27 (−0.52 to −0.02), *P* = 0.027) and stages 1 and 2 (*β* = −0.84 (−1.15 to −0.53), *P* < 0.001). Cognitive performance exhibited significantly lower scores between stages 1 and 2, and stages 2 and 3 for the modified preclinical Alzheimer’s cognitive composite (mPACC; *P* < 0.008) and Mini-Mental State Examination (MMSE; *P* < 0.001).

Similarly, in the TRIAD cohort, Aβ-PET levels showed marked increases between stages 0 and 1 (*β* = 0.63 (0.25 to 1), *P* < 0.001) and stages 1 and 2 (*β* = 1.11 (0.72 to 1.51), *P* < 0.001), before leveling off (*β* = 0.05 (−0.37 to 0.47), *P* = 0.99). MTL tau-PET displayed significant changes between stages 1 and 2 (*β* = 0.71 (0.25 to 1.18), *P* = 0.001), NeoT tau-PET exhibited significant differences between stages 1 and 2 (*β* = 0.73 (0.24 to 1.23), *P* = 0.001), with further elevations at stage 3 (*β* = 0.69 (0.15 to 1.22), *P* = 0.006). Cortical thickness progressively decreased with advancing plasma stages, with significant changes observed between stages 2 and 3 (*β* = −0.66 (−1.24 to −0.07), *P* = 0.022). Cognitive performance showed a trend to higher scores for the Clinical Dementia Rating (CDR) sum of boxes (CDR-SB) and lower for MMSE across plasma stages.

### Plasma tau stages are associated with clinical diagnosis and PET-based stages

**Fig. 3:**
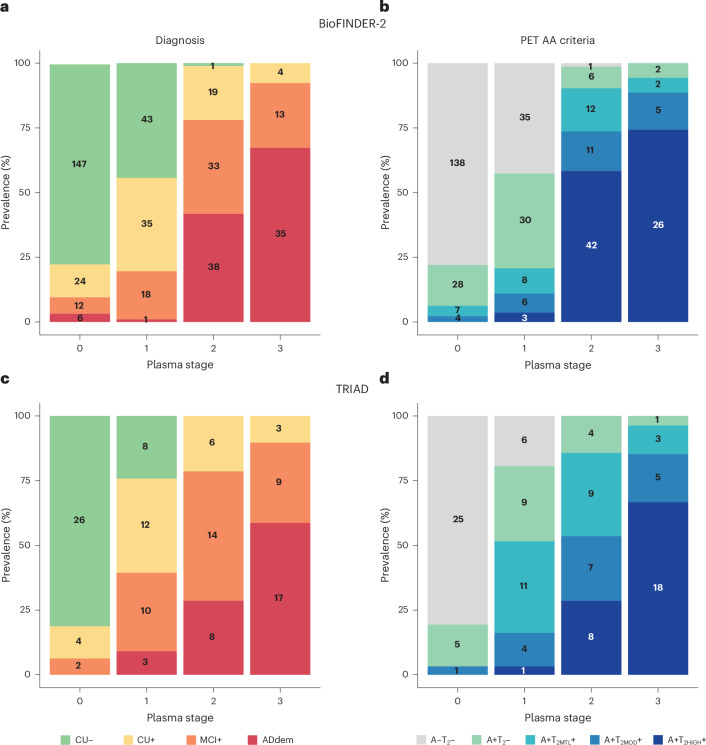
Diagnostic groups and biological staging with the AA criteria classification by plasma stage within the AD continuum*.* **a**–**d**, Associations with diagnostic groups (BioFINDER-2 in **a** and TRIAD in **c**) and with AA criteria (BioFINDER-2 in **b** and TRIAD in **d**). The number of individuals in each group per stage is detailed in the barplots. Individuals with non-AD diagnosis were excluded from these analyses.

Next, we examined how the plasma tau stages were associated with clinical diagnostic groups within the AD continuum. In the BioFINDER-2 cohort (*χ*^2^ = 297, d.f. 9, *P* < 0.001; Fig. 3a), plasma stage 0 predominantly (90%) comprised cognitively unimpaired individuals (Aβ− or Aβ+). In stage 1, this percentage decreased to 80%, with an increase in Aβ+ participants to 56%. Stage 2 primarily consisted of Aβ+ individuals (99%), with 78% showing cognitive impairment (36% MCI+ and 42% ADdem+). By stage 3, 100% of participants were Aβ+, with 92% exhibiting cognitive impairment, including a larger proportion of the dementia group (25% MCI+ and 67% ADdem+).

We then assessed the association between the plasma tau stages and Aβ-and tau-PET imaging stages based on the new AA criteria^28^ (*χ*^2^ = 312.83, d.f. 9, *P* < 0.001; Fig. 3b). In these criteria, individuals are categorized on the basis of their Aβ-PET status (negative or positive) and tau-PET uptake in in MTL and NeoT regions as follows: negative for both amyloid and tau-PET (A−T_2_−), initial (Aβ-PET positive and tau-PET negative, A+T_2_−), early (Aβ-PET positive and tau-PET MTL positive, A+T_2MTL_+), intermediate (Aβ-PET positive and tau-PET MTL and NeoT positive, A+T_2MOD_+) and advanced (Aβ-PET positive and tau-PET MTL and NeoT positive with high uptake in NeoT, A+T_2MOD_+A+T_2HIGH_+). In plasma stage 0, 78% of individuals were Aβ- and tau-PET negative. In stage 1, the negative Aβ- and tau-PET group was reduced to 43%, 37% were Aβ positive and tau-PET negative, and 21% were Aβ- and tau-PET positive. At stage 2, 90% of individuals were tau-PET positive, with 32% classified as early or intermediate and 58% as advanced. In stage 3, 94% of participants were tau-PET positive, with 74% in the advanced category.

These results were replicated in the TRIAD cohort for clinical diagnosis (*χ*^2^ = 109, d.f. 9, *P* < 0.001; Fig. 3c) and for PET AA criteria (*χ*^2^ = 103, d.f. 9, *P* < 0.001; Fig. 3d). In plasma stage 0, most participants (94%) were cognitively unimpaired. This percentage decreased to 61% in stage 1. In stages 2 and 3, 100% of participants were Aβ+, with 79% and 90% being cognitively impaired, respectively. In terms of imaging stages, 81% of individuals in plasma stage 0 were both Aβ- and tau-PET negative. By stage 1, 52% of participants were tau-PET positive. In stage 2, 86% of individuals were tau-PET positive, and by stage 3, tau-PET positivity reached 96%, with 67% classified as having advanced tau-PET uptake.

**Extended Data Fig. 8:**
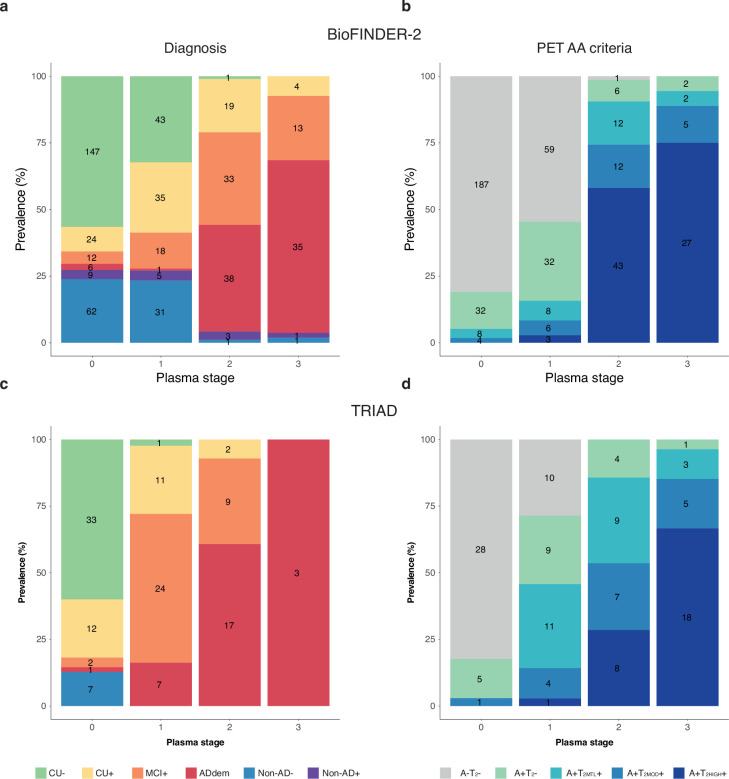
Diagnostic groups and biological staging with the AA criteria classification by plasma stages in all participants in the BioFINDER-2 and TRIAD cohorts. Panels **a** (BioFINDER-2) and **c** (TRIAD) show the association with diagnostic groups, and **b** (BioFINDER-2) and **d** (TRIAD) with the AA criteria. The number of individuals in each group per stage is detailed in the barplots. Only individuals who did not follow the expected biological staging were excluded from PET AA criteria analyses. Abbreviations: AA, Alzheimer’s Association; A-T_2-_, Aβ-negative and tau-negative; A + T_2-_, Aβ-positive and tau-negative; A + T_2MTL+_, Aβ-positive and tau-PET positive in the medial temporal region; A + T_2MOD+,_ Aβ-positive and tau-PET moderate neocortical uptake; A + T_2HIGH+_, Aβ-positive and tau-PET high neocortical uptake; CU-, cognitively unimpaired Aβ-negative; CU + , cognitively unimpaired Aβ-positive; MCI + , mild cognitive impairment Aβ-positive; ADdem, Alzheimer’s disease dementia.

These analyses were also performed including participants outside the AD continuum and are shown in Extended Data Fig. 8.

### Individuals in different plasma tau stages differ in longitudinal trajectories

**Fig. 4:**
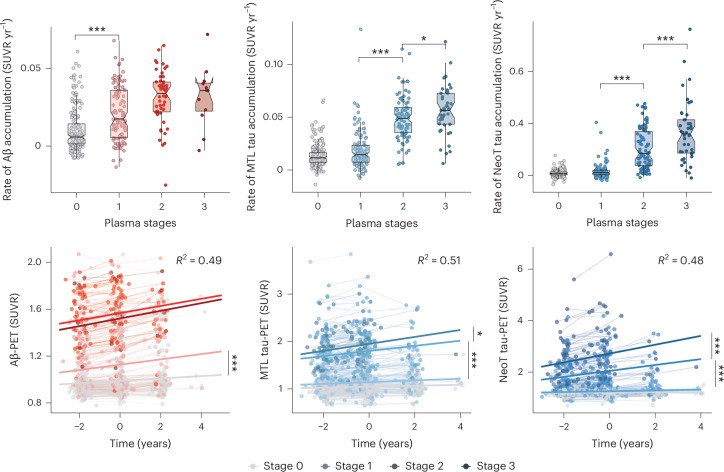
Longitudinal trajectories of AD pathology imaging biomarkers by plasma stage in the BioFINDER-2 cohort. Individual slopes of each biomarker (from unadjusted linear mixed models) are shown in the first row by plasma stage, while actual longitudinal trajectories, colored by plasma stage, are shown in the second row. Colored thick lines represent the mean trajectory per each plasma stage group as calculated with a linear mixed-effect model adjusting for age, sex and *APOE* ε4 carriership. Statistically significant differences (*P* < 0.05, two-sided) on group slopes were extracted from linear mixed models and show differences only by contiguous groups. Boxplots summarize data distribution, showing the median (central line), interquartile range (IQR; box) and whiskers extending to 1.5× the IQR. Plasma draw was considered time 0 for these analyses. Imaging biomarkers were included from all time points available (including before blood draw). We included all individuals with longitudinal data available for each analysis (Aβ-PET: *n* = 368, MTL tau-PET and NeoT tau-PET: *n* = 480). Exact *P* values can be found in Supplementary Table 7. *P < 0.05, ***P < 0.001.

**Fig. 5:**
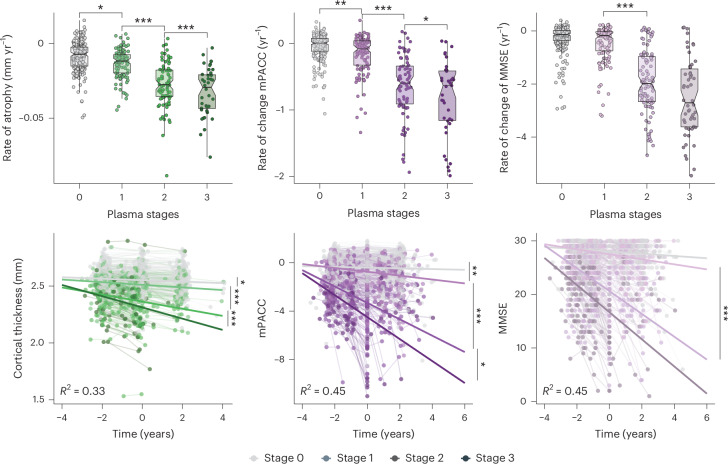
Longitudinal trajectories of atrophy and cognitive decline by plasma stage in the BioFINDER-2 cohort. Individual slopes of each biomarker (from unadjusted linear mixed models) are shown in the first row by plasma stage, while actual longitudinal trajectories, colored by plasma stage, are shown in the second row. Colored thick lines represent the mean trajectory per each stage group as calculated with a linear mixed-effect model adjusting for age, sex and *APOE* ε4 carriership (and years of education for cognitive outcomes). Statistically significant differences (*P* < 0.05, two-sided) on group slopes were extracted from linear mixed models and show differences only by contiguous groups. Individuals with non-AD diagnosis were excluded from these analyses to avoid bias. Boxplots summarize data distribution, showing the median (central line), interquartile range (IQR; box) and whiskers extending to 1.5× the IQR. Plasma draw was considered time 0 for these analyses. Atrophy and cognitive measures were included from all time points available (including before blood draw). We included all individuals with longitudinal data available for each analysis (thickness: *n* = 457, mPACC: *n* = 460 and MMSE: *n* = 491). Exact *P* values can be found in Supplementary Table 7. *P < 0.05, **P < 0.005, ***P < 0.001.

Longitudinal data from the BioFINDER-2 cohort (Supplementary Table 7) were used to examine whether plasma tau stages were associated with longitudinal Aβ-PET and tau-PET uptake—in both MTL and NeoT regions (Fig. 4)—as well as cortical thickness and cognitive decline (Fig. 5).

The evaluation of rate of change in Aβ-PET over time showed that participants in plasma stage 0 accumulated Aβ-PET faster than those in stage 1 (*β*_0vs1_ = 0.06 (0.04 to 0.09), *P* < 0.001). Longitudinal tau-PET of participants in stages 0 and 1 did not reveal any change over time—neither in MTL (*β*_0vs1_ = 0.02 (0.00 to 0.05), *P* = 0.062) nor in NeoT regions (*β*_0vs1_ = 0.02 (−0.01 to 0.05), *P* = 0.122). By contrast, those in stage 2 increased in signal tau-PET significantly faster than those in stage 1 in both regions of interest (MTL: *β*_1vs2_ = 0.11 (0.08 to 0.15), *P* < 0.001; and NeoT: *β*_1vs2_ = 0.23 (0.19 to 0.27), *P* < 0.001), and individuals in stage 3 exhibited an even faster increase in tau-PET signal than those in stage 2 (MTL: *β*_2vs3_ = 0.06 (0.01 to 0.11), *P* = 0.031; and NeoT: *β*_2vs3_ = 0.13 (0.08 to 0.19), *P* < 0.001).

Longitudinal reductions in cortical thickness were observed at every plasma stage, with the rate of decline differing significantly between consecutive stages (*β*_1vs0_ = −0.05 (−0.09 to −0.01), *P* = 0.020; *β*_2vs1_ = −0.19 (−0.24 to −0.13), *P* < 0.001; and *β*_3vs2_ = −0.17 (−0.25 to −0.09), *P* < 0.001). Longitudinal cognitive decline, assessed using mPACC and MMSE scores, demonstrated that higher plasma stages were associated with faster rates of cognitive deterioration in both measures. For mPACC, significant differences were found between successive plasma stages: stage 0 versus stage 1 (*β* = −0.11 (−0.18 to −0.03), *P* = 0.009), stage 1 versus stage 2 (*β* = −0.42 (−0.53 to −0.32), *P* < 0.001) and stage 2 versus stage 3 (*β* = −0.19 (−0.34 to −0.04), *P* = 0.014). For MMSE, significant differences were observed between stage 1 and stage 2 (*β* = −0.50 (−0.60 to −0.41), *P* < 0.001).

## Discussion

In this study, we present a blood-based biomarker staging model for AD using plasma tau biomarkers measured in a single-run MS analysis of a single sample. Our results demonstrate that different plasma tau biomarkers can be used to stage AD biologically in a clinically relevant manner, as we previously showed in CSF^22^. Specifically, we developed a four-stage model based on p-tau217r, p-tau205r and 0N tau. This model was created and tested in one cohort and validated in an independent cohort, showing strong consistency in the distribution of amyloid-PET, tau-PET and neurodegeneration biomarkers, as well as cognitive outcomes, across stages. Furthermore, blood-based stages proved valuable in detecting clinical diagnostic and PET-based stages, as well as associating with the longitudinal progression of the disease, including Aβ- and tau-PET accumulation, atrophy and cognitive decline. Overall, our findings support the utility of distinct blood tau biomarkers for staging AD, offering significant potential for use in both clinical practice and trials.

Previous studies on brain and CSF have suggested that changes in specific phosphorylated and nonphosphorylated tau species occur at distinct moments across AD^9,11^. We first assessed how plasma tau species change along the AD continuum in relation to clinical diagnosis and Aβ status. Our results demonstrate that different plasma tau biomarkers become abnormal at distinct phases of the disease progression. Plasma p-tau217, alongside p-tau231, showed early elevations in cognitively unimpaired individuals transitioning to Aβ positivity. However, p-tau217 exhibited the largest fold changes. Plasma p-tau205 increased later, closer to the onset of symptoms, suggesting it may serve as a more sensitive marker for more advanced disease and accompanying cognitive decline. Nonphosphorylated 0N-tau reached abnormal levels at AD dementia. These findings align with previous studies that identify p-tau217 as the most sensitive plasma biomarker for detecting AD, marked by substantial fold changes and significant longitudinal shifts^12,25,34–37^. Moreover, while primarily observed in CSF^14,15,38^ and to a lesser extent in plasma studies^30,31^, p-tau205 has been more strongly associated with tau-PET imaging and cognitive decline. In addition, brain studies have shown that 0N tau, along with 4R tau, are more prone to aggregation than other isoforms and can be detected in insoluble tau extracts^9,10^. Conversely, not all tau species—such as p-tau199, p-tau202 or the nonphosphorylated tau variants—showed abnormal changes as the disease progressed. Notably, we report that blood levels of the PNS tau isoform remain unaffected by AD progression. Recent research indicates that PNS tau—also known as big tau—does not undergo typical AD-related hyperphosphorylation, is more efficiently ubiquitinated and degraded, has enhanced microtubule-binding capacity and shows a reduced tendency for aggregation^39^. In that study, patients with AD displayed elevated levels of big tau in the pathology-resistant cerebellum, but not in the pathology-sensitive cortex. However, we did not observe such elevated levels in the blood of patients with AD.

Based on these findings, we developed a staging model utilizing p-tau217r, p-tau205r and 0N tau. For p-tau217 and p-tau205, we used the ratios of phosphorylated to nonphosphorylated peptides (tau212-221 and tau195-209, respectively), as we show that these ratios can mitigate the impact of elevated tau levels in individuals with chronic kidney dysfunction, corroborating previous results^33,40^, and had greater consistency across cohorts. The model was also tested using concentrations instead of MS ratios, as they may offer greater applicability and translational potential in clinical settings without greatly diminishing accuracy^41,42^. We identified a consistent pattern of change in Aβ and tau-PET binding, cortical thickness and cognitive decline across cohorts and plasma stages. Aβ-PET primarily exhibited changes between stages 0 and 1, while tau-PET increases were observed between stages 1 and 3. Neurodegeneration markers and cognitive function were characterized by a continuous decrease with advancing stages. This consistency across cohorts was also evident when using plasma stages to predict clinical diagnosis based on cognitive performance and Aβ status. Individuals in plasma stage 0 were mostly cognitively unimpaired, while those in stage 1 were either cognitively unimpaired or MCI. By stage 2, 100% of individuals were Aβ+, with an increased likelihood of cognitive impairment, which further escalated in stage 3. This staging model could serve as a valuable first-line screening tool that, when assessed by a skilled examiner, may complement clinical cognitive diagnosis and improve patient management in clinical settings.

An even more valuable application of this staging system may lie in the capability to detect tau load and spreading in the brain, making it particularly useful for clinical trials and therapeutic interventions. While successful Aβ-targeting immunotherapies have been shown to slow cognitive decline by 25–40% (refs. ^4,43^), their efficacy is reduced in patients with high tau-PET burden^4,5^. A recent study demonstrated that using plasma p-tau217 alone could reduce the number of participants needed for a clinical trial by 75%, and by 94% when combined with tau-PET imaging^44^. This highlights the potential of incorporating plasma p-tau217 into clinical trial workflows to dramatically reduce the need for tau-PET scans, improving efficiency and cutting costs. However, despite plasma p-tau217’s strong ability to identify individuals with elevated tau-PET uptake, its ability to predict continuous tau-PET load is limited^45–48^. By combining biomarkers within a fluid staging system, the process could be further refined, streamlining eligibility workflows and potentially reducing the number of PET scans even further, thus enhancing the overall trial design. In previous studies, we demonstrated that different plasma tau biomarkers are associated with Aβ- and tau-PET imaging^30^ and neuropathological examination^31^ in distinct ways. In this study, we observed a correlation between plasma tau stages and PET-based stages. By plasma stage 0, most individuals were tau-PET negative; in stage 1, most were either tau-PET negative or in early tau-PET stages; by stage 2, individuals were primarily in intermediate stages, and by stage 3, the majority were in advanced stages. However, plasma stages did not perfectly align with imaging stages, which was highlighted as a possibility by the new AA criteria^27,28^. Plasma stages appear to precede imaging stages, which could be explained by two factors: the sensitivity of tau-PET tracers and the differences in the biomarkers being measured. On the one hand, post-mortem studies have shown that a positive [^18^F]flortaucipir tau-PET scan (whether assessed quantitatively or visually) typically reflects tau pathology at Braak stage IV or higher^49^, indicating that the method is not sensitive for detecting very early tau pathology. On the other hand, plasma measures capture soluble tau forms, whereas tau-PET tracers bind to insoluble tau aggregates in the brain^18^. These factors may also indicate that fluid stages are more sensitive in detecting early tau pathology. This is supported by recent data showing that tau-PET outperforms plasma p-tau217 in predicting cognitive decline in symptomatic individuals^50^ but is not significantly higher than plasma p-tau217 in Aβ-negative and Aβ-positive cognitively unimpaired individuals^44^. Further research is required to fully understand the relationship and differences between plasma tau stages and tau imaging stages.

Another significant benefit of an easily accessible staging system lies in its predictive power to determine the expected progression of AD. Our plasma staging model was associated with longitudinal tau-PET accumulation. Individuals classified as stage 0 or stage 1 showed no evidence of an increase in tau-PET signal over time, whereas those in stages 2 and 3 demonstrated significant increases. Interestingly, the rate of tau-PET signal increases in the medial temporal lobe was similar between stages 2 and 3, whereas neocortical temporal regions exhibited more pronounced differences in tau-PET uptake between these stages. This suggests that individuals in stage 2 will continue to accumulate tau aggregates in both brain regions, while those in stage 3 may have reached the maximum accumulation in the medial temporal lobe but will continue to accumulate tau in later accumulation areas. This information could be crucial for identifying patients who may benefit from treatment, especially in the context of future anti-tau therapies, where predicting tau spread in the brain will be critical. In addition, each plasma stage was associated with a different mean rate of cognitive decline. This capability could assist clinicians to forecast individual deterioration based on plasma stage. For example, the efficacy of anti-amyloid therapies to delay cognitive decline has been suggested to be affected not only by tau-PET levels, but also cognitive status. Individuals with same low-to-medium tau-PET burden but more advanced clinical dementia ratings may have fewer additional months of independence with the same treatment^51^.

The main strengths of this study include the simultaneous measurement of multiple plasma tau species in a single analysis, allowing us to identify which biomarkers are most effective for staging AD and to develop a staging system that reflects various pathological changes. In addition, this model was validated across two independent cohorts, with the inclusion of longitudinal data further supporting its robustness. However, this study is not without limitations. We observed a late increase in the levels of p-tau181 compared with previous reports, probably due to differences in the specific target measured between MS and immunoassay-based methods, as described^30^. In addition, while biofluid concentrations represent a snapshot of the balance between production and clearance of specific proteins at a given time, imaging biomarkers reflect the cumulative aggregation of pathology over time. As a result, direct comparisons between fluid and imaging biomarkers may not always be precise. Moreover, PET imaging acquisition in the two cohorts was conducted using different methodologies (that is, scanners and tracers), which may have influenced the classification of PET status and, consequently, the relationship between imaging stages and plasma stages. In addition, because the blood data were not acquired at baseline but at the second visit of the BioFINDER-2 cohort, the available follow-up data were insufficient to perform purely prospective analyses. Therefore, we also included retrospective data in our longitudinal analyses. In this regard, time-to-cognitive decline analysis could not be performed due to the limited availability of prospective longitudinal data. We acknowledge the potential limitations and biases introduced by this approach, and further research is needed to validate these findings. Furthermore, the results were evaluated at the group level, limiting direct clinical application. In this regard, while the staging system may be effective at the group level, further work is needed to improve its specificity for potential clinical use. Future studies should validate the staging model in cohorts with post-mortem neuropathological examination and assess whether incorporating additional blood biomarkers could enhance the accuracy of the plasma staging system. Moreover, research involving more diverse cohorts, particularly those with a focus on peripheral comorbidities, is needed to evaluate how these might influence the model. Finally, standardization of the staging model across various settings—through the establishment of cutoffs, common reference materials and validation using different technologies—will be essential to advance this model toward clinical implementation.

In conclusion, we have developed and validated a biomarker-based staging model using plasma tau biomarkers from a single sample and analysis. Our results demonstrate that distinct plasma tau biomarkers can accurately stage AD, offering valuable insights for identifying clinical diagnostic and imaging stages, as well as predicting longitudinal tau-PET accumulation, neurodegeneration and cognitive decline. We believe these findings will enhance the potential application of blood tau biomarkers, improving patient management in both clinical trials and routine clinical practice.

## Methods

### Participants

#### BioFINDER

This study included participants from the BioFINDER-2 (NCT03174938) cohort covering the whole AD continuum from preclinical disease to dementia. Cognitively unimpaired individuals were recruited from a population-based study in Malmö, Sweden, as previously described^52^. These participants did not meet the criteria for mild cognitive impairment (MCI) or dementia at baseline. In addition, patients with subjective cognitive decline, MCI and AD dementia were recruited from the memory clinics of Skåne University Hospital and Ängelholm hospital in Sweden. All participants provided written informed consent and were recruited consecutively without excluding any eligible participants between March 2019 and November 2022. MCI was defined using a broad neuropsychological battery and dementia according to the Diagnostic and Statistical Manual of Mental Disorders (DSM-5) criteria for major neurocognitive disorder, as previously described^52,53^. Patients with MCI or dementia were required to be Aβ positive—determined by either CSF or PET—to be considered as having AD. Participants with other etiological diagnosis than AD were classified as non-AD and are referred as cognitively impaired not due to AD. General cognition was assessed using the MMSE and memory using the ten-word delayed recall test from the Alzheimer’s Disease Assessment Scale-Cognitive subscale^54^. A modified preclinical Alzheimer cognitive composite was used to assess cognitive decline sensitive to the earliest changes^55^, as previously described^22^. The study was approved by the Regional Ethics Committee in Lund, Sweden (Dnr 2016-1053).

#### TRIAD

This study assessed individuals from the TRIAD^56^ cohort. TRIAD patients were approached consecutively, and no eligible participants were excluded. Data from the TRIAD study were collected between October 2019 and May 2024. All participants provided written informed consent and had clinical evaluations by dementia specialists, plasma assessments and structural magnetic resonance imaging (MRI), as well as amyloid-PET with [^18^F]AZD4694 and tau-PET with [^18^F]MK6240. Evaluations of participants included a review of their medical history and an interview with the participant and their study partner, a neurological examination by a dementia specialist and a detailed neuropsychological examination. Participants were approached consecutively, and data were collected prospectively. Cognitively unimpaired individuals had no objective cognitive impairment and a CDR score of 0. Individuals with MCI had subjective and/or objective cognitive impairment and a CDR score of 0.5. Individuals with dementia had a CDR 1 or greater. Patients with MCI or dementia had to be Aβ positive by either CSF or PET to be considered due to AD. Patients with cognitive impairment but negative Aβ status were classified as cognitively impaired not due to AD. Participants were excluded from this study if they had systemic conditions that were not adequately controlled through a stable medication regimen. Other exclusion criteria were active substance abuse, recent head trauma, recent major surgery and MRI and PET safety contraindications. The study was approved by the Montreal Neurological Institute PET working committee and the Douglas Mental Health University Institute Research Ethics Board (ethical approval: MP-18-2019-223, IUSMD-19-05).

### Plasma tau MS analysis

Available plasma samples were analyzed by MS detection of phosphorylated and nonphosphorylated tau peptides following a previously established protocol^21,30^. In summary, 1 ml of plasma samples (collected with EDTA) were thawed, vortexed at 2,000 rpm for 30 s and centrifuged for 10 min at 4,000*g*. Tau protein was isolated via immunoprecipitation with a combination of three antibodies (Tau12, purified anti-tau, 6–18 antibody, 806501, BioLegend; HT7, tau monoclonal antibody, MN1000, Thermofisher; and BT2, tau monoclonal antibody, MN1010, Thermofisher), followed by enrichment using perchloric acid precipitation and desalting. The samples were then subjected to tryptic digestion by adding trypsin solution (sequencing grade, Promega) at 0.1 µg per sample (final concentration of 2.5 µg ml^−1^ in 50 mM ammonium bicarbonate) and incubating at 37 °C overnight. After 18 h, the digestion was halted by adding trifluoroacetic acid to a final concentration of 0.1%. The samples were then lyophilized and stored at −20 °C until MS analysis. For MS analysis, a hybrid Orbitrap mass spectrometer (Lumos, Thermo Scientific) equipped with an EasySpray nano-ESI ion source was used. The instrument was operated in positive ion mode with the following settings for parallel reaction monitoring scans: higher-energy collisional dissociation activation, Orbitrap detector, 60,000 resolution, scan range of 250–1,200 *m*/*z*, radio frequency lens at 30%, and Easy-IC enabled. Quadrupole isolation was applied with a 0.7 *m*/*z* window. Parameters such as maximum injection time, normalized automatic gain control target, optimal collision energy and field asymmetric ion mobility spectrometry voltage were optimized for each peptide. Details of the endogenous tryptic peptides analyzed are provided in Supplementary Table 8. Liquid chromatography–MS data acquisition was performed using Xcalibur 4.5 and Tune 3.5 software (Thermo Scientific), and the data were analyzed with Skyline (McCoss Lab, University of Washington). The analysis of plasma samples was conducted without knowledge of participant information.

### Imaging acquisition and processing

In BioFINDER-2, imaging procedures have been described previously^22^. In brief, Aβ-PET and tau-PET were acquired using [^18^F]flutemetamol and [^18^F]RO948, respectively. Amyloid-PET binding was measured as the standardized uptake value ratio (SUVR) using a neocortical meta-region of interest (ROI) and with whole cerebellum as a reference region. Of note, most of the patients with AD dementia did not undergo amyloid-PET in BioFINDER-2 owing to the study design. Tau-PET binding was measured in regions covering early (MTL) and intermediate (NeoT) tau deposition areas^57^. For assessing cortical thickness, T1-weighted anatomical magnetization-prepared rapid gradient echo (MPRAGE) images (1-mm isotropic voxels) were used. A cortical thickness meta-ROI was calculated including entorhinal, inferior temporal, middle temporal and fusiform using FreeSurfer (version 6.0; https://surfer.nmr.mgh.harvard.edu) parcellation, which are areas known to be susceptible to AD-related atrophy^58^. In TRIAD, [^18^F]AZD4694 PET and [^18^F]MK6240 PET scans were acquired with a brain-dedicated Siemens High Resolution Research Tomograph. [^18^F]AZD4694 PET images were acquired 40–70 min after bolus injection and reconstructed on a four-dimensional volume with three frames (3 × 600 s), as previously described^59^. [^18^F]MK6240 PET images were acquired at 90–110 min after bolus radiotracer injection and reconstructed on a four-dimensional volume with four frames (4 × 300 s) (ref. ^60^). A 6-min transmission scan with a rotating ^137^Cs point source followed each PET acquisition for attenuation correction. PET images were corrected for decay, motion, dead time, random and scattered coincidences. T1-weighted MRIs were acquired at the Montreal Neurological Institute on a 3 T Siemens Magnetom using a standard head coil. They underwent correction for nonuniformity and field distortion and were processed using an in-house pipeline. PET images were automatically registered to the T1-weighted image space, and the T1-weighted images were linearly and nonlinearly registered to the Montreal Neurological Institute (MNI) reference space. To minimize interference of meningeal spillover, [^18^F]MK6240 images were stripped of meningeal signal in native space before spatial normalization and smoothing^61^. [^18^F]AZD4694 SUVR maps were calculated using the whole cerebellum gray matter as the reference region, and [^18^F]MK6240 SUVR maps were generated using the inferior cerebellar gray matter as a reference region. Spatial smoothing allowed the PET images to achieve an 8-mm full-width at half-maximum resolution. Amyloid-β SUVR from a neocortical ROI for each participant was estimated by averaging the SUVR from the precuneus, prefrontal, orbitofrontal, parietal, temporal and cingulate cortices^59^, with amyloid-β positivity defined as an [^18^F]AZD4694 above 1.55 (centiloids = 24). The SUVR from the temporal meta-ROI, a composite mask commonly used as a summary measure of tau-PET, was calculated from the entorhinal, parahippocampal, amygdala, fusiform, inferior and middle temporal cortices, as previously described^62^, with positivity defined as SUVRs above 1.24 (ref. ^63^). T1-weighted MRIs were acquired at the MNI on a 3 T Siemens Magnetom scanner using a standard head coil. A composite neurodegeneration summary measure was used, comprising a surface-area weighted average of the mean cortical thickness in the following individual ROIs bilaterally: entorhinal, inferior temporal, middle temporal and fusiform as previously described^62^. Cortical thickness measures were derived from FreeSurfer (v7.4).

### Statistics and reproducibility

First, we plotted each plasma biomarker against clinical diagnosis using CU− as the control group to create *z* scores of each biomarker. Individuals with non-AD diagnoses were excluded from this analysis. Differences across diagnoses were calculated using analysis of covariance (ANCOVA) adjusted by age and sex and *APOE* ε4 carriership followed by Tukey’s corrected, post-hoc pairwise comparisons (two-sided analysis). Those biomarkers that showed differences across the consecutive diagnosis groups were selected for creating the staging model (that is, p-tau217r, p-tau205r and 0N-tau).

#### Creation of the model

Each participant from the BioFINDER-2 cohort was characterized by these three main biomarkers as a point in a three-dimensional space, in which each biomarker corresponded to an orthogonal axis (Extended Data Fig. 2). We then divided the whole BioFINDER-2 cohort into ten partitions (or ten folds). We used 9/10 parts of the sample to train a *k*-means clustering approach that aimed to divide the sample into four groups (or stages) using the clusterboot function (fpc package, v 2.2-11). We applied the resulting model to the test data (1/10 of the BioFINDER-2 cohort) and the validation set (TRIAD cohort). This was repeated for each of the ten parts of the BioFINDER-2 cohort, so the model was created in a completely independent sample from the one it was tested. This approach was repeated five times with different initialization parameters to avoid randomness in the model creation and application. The final stage for each individual was assessed as the mode of the five repetitions in the BioFINDER-2 individuals and all the classifications (50) of the TRIAD participants. For testing replicability, we looked at how many times each individual was classified in the same stage with 5,000 different initializations.

#### Cross-sectional assessment of the plasma staging model

Next, we compared the groups created with the clustering approach to other biomarkers and disease scales cross-sectionally. Statistical differences by plasma stage were assessed using ANCOVA adjusted by age and sex and *APOE* ε4 carriership and years of education for cognitive outcomes followed by Tukey’s corrected, post-hoc pairwise comparisons (two-sided analysis). These analyses were performed for BioFINDER-2 and TRIAD participants independently.

#### Longitudinal assessment of the plasma staging model

Finally, we also performed longitudinal analyses to assess tau-PET accumulation, atrophy and cognitive decline in the BioFINDER-2 cohort. To this aim, we used linear mixed models (lme4 package, v1.1-34) with tau, cognition or cortical thickness as the outcome and the interaction between time and plasma stages as the predictor, with random slopes and intercepts adjusting for age and sex and *APOE* ε4 carriership (and years of education for cognitive outcomes). Importantly, all available outcome data were used to calculate these changes, including data from before and after the acquisition of the plasma samples (−2 to 4 years before/after plasma sampling).

All analyses were performed with R (version 4.3.1). Two-sided *P* values less than 0.05 were considered statistically significant. For comparisons between plasma stages (that is, biomarker levels and rates of change), false discovery rate correction was applied to account for multiple comparisons. All plots were done with the ggplot2 package (v3.5.1).

### Reporting summary

Further information on research design is available in the Nature Portfolio Reporting Summary linked to this article.

## Supplementary information

Supplementary Information Supplementary Tables 1–8.

Reporting Summary

Peer Review File

## Data Availability

This study includes no data deposited in external repositories. Anonymized data can be shared upon reasonable request from a qualified academic investigator, for the sole purpose of replicating procedures and results presented in the Article, as long as data transfer agrees with local legislation and with the local ethical review board of each cohort, which must be regulated in a material/data transfer agreement. Researchers interested in accessing the datasets should contact the corresponding authors and provide a brief research proposal outlining the intended use of the data. Data requests will be evaluated on the basis of scientific merit and compliance with ethical and legal requirements. Cohort-specific guidelines: BioFINDER-2 (https://biofinder.se) and TRIAD (https://triad.tnl-mcgill.com).

## Code availability

The code used for the analyses in this study is available from the corresponding authors upon request. Interested researchers are encouraged to contact the corresponding authors with a brief description of the intended use. Access will be provided for noncommercial purposes and solely for reproducing the results presented in this Article.

## References

[1] 2024 Alzheimer’s disease facts and figures. *Alzheimers Dement.* **20**, 3708–3821 (2024). 10.1002/alz.13809 PMC11095490 38689398

[2] Gonnella, J. S., Hornbrook, M. C. & Louis, D. Z. Staging of disease. A case-mix measurement. *JAMA* **251**, 637–644 (1984). 6418903

[3] Therriault, J. et al. Biomarker-based staging of Alzheimer disease: rationale and clinical applications. *Nat. Rev. Neurol.*10.1038/s41582-024-00942-2 (2024). 10.1038/s41582-024-00942-2 38429551

[4] Sims, J. R. et al. Donanemab in early symptomatic Alzheimer disease: the TRAILBLAZER-ALZ 2 randomized clinical trial. *JAMA* **330**, 512–527 (2023). 37459141 10.1001/jama.2023.13239 PMC10352931

[5] Pontecorvo, M. J. et al. Association of donanemab treatment with exploratory plasma biomarkers in early symptomatic Alzheimer disease: a secondary analysis of the TRAILBLAZER-ALZ randomized clinical trial. *JAMA Neurol.* **79**, 1250–1259 (2022). 36251300 10.1001/jamaneurol.2022.3392 PMC9577883

[6] Hansson, O. et al. The Alzheimer’s Association appropriate use recommendations for blood biomarkers in Alzheimer’s disease. *Alzheimers Dement.* **18**, 2669–2686 (2022). 35908251 10.1002/alz.12756 PMC10087669

[7] Hansson, O., Blennow, K., Zetterberg, H. & Dage, J. Blood biomarkers for Alzheimer’s disease in clinical practice and trials. *Nat. Aging* **3**, 506–519 (2023). 37202517 10.1038/s43587-023-00403-3 PMC10979350

[8] Therriault, J. et al. Biomarker modeling of Alzheimer’s disease using PET-based Braak staging. *Nat. Aging* **2**, 526–535 (2022). 37118445 10.1038/s43587-022-00204-0 PMC10154209

[9] Wesseling, H. et al. Tau PTM profiles identify patient heterogeneity and stages of Alzheimer’s disease. *Cell* **183**, 1699–1713.e13 (2020). 33188775 10.1016/j.cell.2020.10.029 PMC8168922

[10] Lantero-Rodriguez, J. et al. Tau protein profiling in tauopathies: a human brain study. *Mol. Neurodegener.* **19**, 54 (2024). 39026372 10.1186/s13024-024-00741-9 PMC11264707

[11] Barthélemy, N. R. et al. A soluble phosphorylated tau signature links tau, amyloid and the evolution of stages of dominantly inherited Alzheimer’s disease. *Nat. Med.* **26**, 398–407 (2020). 32161412 10.1038/s41591-020-0781-z PMC7309367

[12] Milà-Alomà, M. et al. Plasma p-tau231 and p-tau217 as state markers of amyloid-β pathology in preclinical Alzheimer’s disease. *Nat. Med.*10.1038/s41591-022-01925-w (2022). 10.1038/s41591-022-01925-w PMC9499867 35953717

[13] Suárez-Calvet, M. et al. Novel tau biomarkers phosphorylated at T181, T217 or T231 rise in the initial stages of the preclinical Alzheimer’s continuum when only subtle changes in Aβ pathology are detected. *EMBO Mol. Med.* **12**, e12921 (2020). 33169916 10.15252/emmm.202012921 PMC7721364

[14] Lantero-Rodriguez, J. et al. CSF p-tau205: a biomarker of tau pathology in Alzheimer’s disease. *Acta Neuropathol.* **147**, 12 (2024). 38184490 10.1007/s00401-023-02659-w PMC10771353

[15] Barthélemy, N. R. et al. CSF tau phosphorylation occupancies at T217 and T205 represent improved biomarkers of amyloid and tau pathology in Alzheimer’s disease. *Nat. Aging* **3**, 391–401 (2023). 37117788 10.1038/s43587-023-00380-7 PMC10154225

[16] Lantero-Rodriguez, J. et al. Plasma and CSF concentrations of N-terminal tau fragments associate with in vivo neurofibrillary tangle burden. *Alzheimers Dement.* **19**, 5343–5354 (2023). 37190913 10.1002/alz.13119

[17] Lantero-Rodriguez, J. et al. Plasma N-terminal containing tau fragments (NTA-tau): a biomarker of tau deposition in Alzheimer’s disease. *Mol. Neurodegener.* **19**, 19 (2024). 38365825 10.1186/s13024-024-00707-x PMC10874032

[18] Mattsson-Carlgren, N. et al. Aβ deposition is associated with increases in soluble and phosphorylated tau that precede a positive Tau PET in Alzheimer’s disease. *Sci. Adv.* **6**, eaaz2387 (2020). 32426454 10.1126/sciadv.aaz2387 PMC7159908

[19] Horie, K. et al. CSF tau microtubule-binding region identifies pathological changes in primary tauopathies. *Nat. Med.*10.1038/s41591-022-02075-9 (2022). 10.1038/s41591-022-02075-9 PMC9800273 36424467

[20] Horie, K. et al. CSF MTBR-tau243 is a specific biomarker of tau tangle pathology in Alzheimer’s disease. *Nat. Med.* **29**, 1954–1963 (2023). 37443334 10.1038/s41591-023-02443-z PMC10427417

[21] Gobom, J. et al. Antibody-free measurement of cerebrospinal fluid tau phosphorylation across the Alzheimer’s disease continuum. *Mol. Neurodegener.* **17**, 81 (2022). 36510321 10.1186/s13024-022-00586-0 PMC9743664

[22] Salvadó, G. et al. Disease staging of Alzheimer’s disease using a CSF-based biomarker model. *Nat. Aging* **4**, 694–708 (2024). 38514824 10.1038/s43587-024-00599-y PMC11108782

[23] Karikari, T. K. et al. Blood phosphorylated tau 181 as a biomarker for Alzheimer’s disease: a diagnostic performance and prediction modelling study using data from four prospective cohorts. *Lancet Neurol.* **19**, 422–433 (2020). 32333900 10.1016/S1474-4422(20)30071-5

[24] Ashton, N. J. et al. Plasma p-tau231: a new biomarker for incipient Alzheimer’s disease pathology. *Acta Neuropathol.* **141**, 709–724 (2021). 33585983 10.1007/s00401-021-02275-6 PMC8043944

[25] Mattsson-Carlgren, N. et al. Soluble P-tau217 reflects amyloid and tau pathology and mediates the association of amyloid with tau. *EMBO Mol. Med.* **13**, e14022 (2021). 33949133 10.15252/emmm.202114022 PMC8185545

[26] Salvadó, G. et al. Specific associations between plasma biomarkers and postmortem amyloid plaque and tau tangle loads. *EMBO Mol. Med.* **15**, e17123 (2023). 36912178 10.15252/emmm.202217123 PMC10165361

[27] Jack, C. R. Jr et al. Revised criteria for the diagnosis and staging of Alzheimer’s disease. *Nat. Med.* **30**, 2121–2124 (2024). 38942991 10.1038/s41591-024-02988-7 PMC11630478

[28] Jack, C. R. Jr et al. Revised criteria for diagnosis and staging of Alzheimer’s disease: Alzheimer’s Association Workgroup. *Alzheimers Dement.* **20**, 5143–5169 (2024). 10.1002/alz.13859 PMC11350039 38934362

[29] Jack, C. R. Jr et al. NIA-AA Research Framework: toward a biological definition of Alzheimer’s disease. *Alzheimers Dement.* **14**, 535–562 (2018). 29653606 10.1016/j.jalz.2018.02.018 PMC5958625

[30] Montoliu-Gaya, L. et al. Mass spectrometric simultaneous quantification of tau species in plasma shows differential associations with amyloid and tau pathologies. *Nat. Aging*10.1038/s43587-023-00405-1 (2023). 10.1038/s43587-023-00405-1 PMC10275761 37198279

[31] Montoliu-Gaya, L. et al. Optimal blood tau species for the detection of Alzheimer’s disease neuropathology: an immunoprecipitation mass spectrometry and autopsy study. *Acta Neuropathol.* **147**, 5 (2023). 38159140 10.1007/s00401-023-02660-3 PMC10757700

[32] Fischer, I. & Baas, P. W. Resurrecting the mysteries of big tau. *Trends Neurosci.* **43**, 493–504 (2020). 32434664 10.1016/j.tins.2020.04.007 PMC7999525

[33] Janelidze, S., Barthélemy, N. R., He, Y., Bateman, R. J. & Hansson, O. Mitigating the associations of kidney dysfunction with blood biomarkers of Alzheimer disease by using phosphorylated tau to total tau ratios. *JAMA Neurol.* **80**, 516–522 (2023). 10.1001/jamaneurol.2023.0199 PMC10061310 36987840

[34] Palmqvist, S. et al. Discriminative accuracy of plasma phospho-tau217 for Alzheimer disease vs other neurodegenerative disorders. *JAMA* **324**, 772–781 (2020). 32722745 10.1001/jama.2020.12134 PMC7388060

[35] Mattsson-Carlgren, N. et al. Longitudinal plasma p-tau217 is increased in early stages of Alzheimer’s disease. *Brain* **143**, 3234–3241 (2020). 33068398 10.1093/brain/awaa286 PMC7719022

[36] Janelidze, S. et al. Associations of plasma phospho-tau217 levels with tau positron emission tomography in early Alzheimer disease. *JAMA Neurol.* **78**, 149–156 (2021). 33165506 10.1001/jamaneurol.2020.4201 PMC7653537

[37] Ashton, N. J. et al. Differential roles of Aβ42/40, p-tau231 and p-tau217 for Alzheimer’s trial selection and disease monitoring. *Nat. Med.* **28**, 2555–2562 (2022). 36456833 10.1038/s41591-022-02074-w PMC9800279

[38] Strain, J. F. et al. CSF tau phosphorylation at Thr205 is associated with loss of white matter integrity in autosomal dominant Alzheimer disease. *Neurobiol. Dis.* **168**, 105714 (2022). 35358703 10.1016/j.nbd.2022.105714 PMC9701560

[39] Chung, D. C. et al. The big tau splice isoform resists Alzheimer’s-related pathological changes. Preprint at *bioRxiv*10.1101/2024.07.30.605685 (2024).

[40] Mielke, M. M. et al. Comparison of plasma phosphorylated tau species with amyloid and tau positron emission tomography, neurodegeneration, vascular pathology, and cognitive outcomes. *JAMA Neurol.* **78**, 1108–1117 (2021). 34309632 10.1001/jamaneurol.2021.2293 PMC8314178

[41] Palmqvist, S. et al. Blood biomarkers to detect Alzheimer disease in primary care and secondary care. *JAMA* **332**, 1245–1257 (2024). 39068545 10.1001/jama.2024.13855 PMC11284636

[42] Barthélemy, N. R. et al. Highly accurate blood test for Alzheimer’s disease is similar or superior to clinical cerebrospinal fluid tests. *Nat. Med.* **30**, 1085–1095 (2024). 38382645 10.1038/s41591-024-02869-z PMC11031399

[43] van Dyck, C. H. et al. Lecanemab in early Alzheimer’s disease. *N. Engl. J. Med.* **388**, 9–21 (2023). 36449413 10.1056/NEJMoa2212948

[44] Ossenkoppele, R. et al. Plasma p-tau217 and tau-PET predict future cognitive decline among cognitively unimpaired individuals: implications for clinical trials. *Nat. Aging* **5**, 883–896 (2025). 40155777 10.1038/s43587-025-00835-z PMC12092243

[45] Mattsson-Carlgren, N. et al. Plasma biomarker strategy for selecting patients with Alzheimer disease for antiamyloid immunotherapies. *JAMA Neurol.* **81**, 69–78 (2024). 38048096 10.1001/jamaneurol.2023.4596 PMC10696515

[46] Feizpour, A. et al. Detection and staging of Alzheimer’s disease by plasma pTau217 on a high throughput immunoassay platform. *EBioMedicine* **109**, 105405 (2024). 39437657 10.1016/j.ebiom.2024.105405 PMC11536028

[47] Sperling, R. A. et al. Amyloid and tau prediction of cognitive and functional decline in unimpaired older individuals: longitudinal data from the A4 and LEARN Studies. *J. Prev. Alzheimers Dis.* **11**, 802–813 (2024). 39044488 10.14283/jpad.2024.122 PMC11266444

[48] Doré, V. et al. Plasma p217+tau versus NAV4694 amyloid and MK6240 tau PET across the Alzheimer’s continuum. *Alzheimers Dement.* **14**, e12307 (2022). 10.1002/dad2.12307 PMC8984092 35415202

[49] Freiburghaus, T. et al. Association of in vivo retention of [^18^F] flortaucipir PET with tau neuropathology in corresponding brain regions. *Acta Neuropathol.* **148**, 44 (2024). 39297933 10.1007/s00401-024-02801-2 PMC11413084

[50] Smith, R. et al. Tau-PET is superior to phospho-tau when predicting cognitive decline in symptomatic AD patients. *Alzheimers Dement.* **19**, 2497–2507 (2023). 36516028 10.1002/alz.12875 PMC10264552

[51] Hartz, S. M. et al. Assessing the clinical meaningfulness of slowing CDR-SB progression with disease-modifying therapies for Alzheimer’s disease. *Alzheimers Dement.* **11**, e70033 (2025). 10.1002/trc2.70033 PMC11822626 39949872

[52] Quadalti, C. et al. Clinical effects of Lewy body pathology in cognitively impaired individuals. *Nat. Med.* **29**, 1964–1970 (2023). 37464058 10.1038/s41591-023-02449-7 PMC10427416

[53] Palmqvist, S. et al. Cognitive effects of Lewy body pathology in clinically unimpaired individuals. *Nat. Med.* **29**, 1971–1978 (2023). 37464059 10.1038/s41591-023-02450-0 PMC10427420

[54] Rosen, W. G., Mohs, R. C. & Davis, K. L. A new rating scale for Alzheimer’s disease. *Am. J. Psychiatry* **141**, 1356–1364 (1984). 6496779 10.1176/ajp.141.11.1356

[55] Donohue, M. C. et al. The preclinical Alzheimer cognitive composite: measuring amyloid-related decline. *JAMA Neurol.* **71**, 961–970 (2014). 24886908 10.1001/jamaneurol.2014.803 PMC4439182

[56] Therriault, J. et al. Association of apolipoprotein E ε4 with medial temporal tau independent of amyloid-β. *JAMA Neurol.* **77**, 470–479 (2020). 31860000 10.1001/jamaneurol.2019.4421 PMC6990684

[57] Ossenkoppele, R. et al. Amyloid and tau PET-positive cognitively unimpaired individuals are at high risk for future cognitive decline. *Nat. Med.* **28**, 2381–2387 (2022). 36357681 10.1038/s41591-022-02049-x PMC9671808

[58] Jack, C. R. Jr et al. Different definitions of neurodegeneration produce similar amyloid/neurodegeneration biomarker group findings. *Brain* **138**, 3747–3759 (2015). 26428666 10.1093/brain/awv283 PMC4655341

[59] Therriault, J. et al. Determining amyloid-β positivity using ^18^F-AZD4694 PET imaging. *J. Nucl. Med.* **62**, 247–252 (2021). 32737243 10.2967/jnumed.120.245209

[60] Therriault, J. et al. Frequency of biologically defined Alzheimer disease in relation to age, sex, APOE ε4, and cognitive impairment. *Neurology* **96**, e975–e985 (2021). 33443136 10.1212/WNL.0000000000011416 PMC8055338

[61] Pascoal, T. A. et al. ^18^F-MK-6240 PET for early and late detection of neurofibrillary tangles. *Brain* **143**, 2818–2830 (2020). 32671408 10.1093/brain/awaa180

[62] Jack, C. R. Jr et al. Defining imaging biomarker cut points for brain aging and Alzheimer’s disease. *Alzheimers Dement.* **13**, 205–216 (2017). 27697430 10.1016/j.jalz.2016.08.005 PMC5344738

[63] Therriault, J. et al. Intrinsic connectivity of the human brain provides scaffold for tau aggregation in clinical variants of Alzheimer’s disease. *Sci. Transl. Med.* **14**, eabc8693 (2022). 36001678 10.1126/scitranslmed.abc8693

