# Supplementary material for: Plasma tau biomarkers for biological staging of Alzheimer’s disease
Source: Nat Aging. 2025 Aug 22;5(11):2297–308. doi: 10.1038/s43587-025-00951-w (PMC12618263; doi:10.1038/s43587-025-00951-w)
Supplement: Supplementary file 1 — Supplementary Tables 1–8. [file 43587_2025_951_MOESM1_ESM.pdf]

nature aging

Supplementary information https://doi.org/10.1038/s43587-025-00951-w

Plasma tau biomarkers for biological staging of Alzheimer's disease

In the format provided by the authors and unedited

Supplementary Table 1. Differences in plasma biomarkers by diagnosis within the Alzheimer´s Disease (AD) continuum.

Abbreviations: CU-, cognitively unimpaired Aβ-negative; CU+, cognitively unimpaired Aβ-positive; FDR, false discovery rate; MCI+, mild cognitive impairment Aβ-positive; ADdem, Alzheimer's disease dementia.

	CU- vs. CU+		CU+ vs. MCI+		MCI+ vs. ADdem+	
**Biomarker**	Standard β	p-value	Standard β	p-value	Standard β	p-value
**p-tau205_r**	-0.43	0.001	-0.57	<0.001	-0.49	<0.001
**p-tau217_r**	-0.74	<0.001	-0.54	<0.001	-0.71	<0.001
**p-tau181**	-0.43	0.001	-0.28	0.143	-0.59	<0.001
**Tau 212-221**	0.07	0.964	-0.2	0.569	-0.15	0.776
**Tau 195-209**	-0.13	0.759	-0.28	0.233	-0.31	0.156
**p-tau199**	-0.11	0.851	-0.11	0.904	-0.28	0.278
**p-tau202**	0.01	1	-0.12	0.887	-0.24	0.452
**p-tau231**	-0.63	<0.001	-0.39	0.005	-0.63	<0.001
**Tau-0N**	-0.35	0.031	-0.24	0.335	-0.53	0.001
**Tau-1N**	-0.08	0.943	-0.12	0.846	-0.38	0.067
**PNS-tau 131-138**	0.22	0.407	-0.19	0.642	-0.13	0.842
**PNS-tau 275-291**	0.07	0.954	-0.08	0.964	-0.07	0.971

Supplementary Table 2. Effect of CKD on blood biomarker levels.

Participants were classified based on their eGFR levels (positivity: <60 mL/min/1.73m^2). CKD, chronic kidney disease.

Biomarker	Estimate_ CKDneg - CKDpos	p-value_ CKDneg - CKDpos
p-tau205_r	-0.09	0.377
p-tau217_r	0.04	0.688
p-tau181	-0.62	<0.001
Tau 212-221	-0.60	<0.001
p-tau217	-0.20	0.056
Tau 195-209	-0.67	<0.001
p-tau199	-0.59	<0.001
p-tau202	-0.46	<0.001
p-tau205	-0.42	<0.001
p-tau231	-0.48	<0.001
Tau-0N	-0.31	0.003
Tau-1N	-0.48	<0.001
PNS-tau 131-138	-0.36	0.001
PNS-tau 275-291	-0.34	0.002

Supplementary Table 3. Percentage of individuals that were placed in the same plasma-based stage using different model initializations.

BioFINDER-2		TRIAD	
% of iterations in the same plasma-stage	% of individuals	% of iterations in the same plasma-stage	% of iterations in the same plasma-stage
40-50%	0.2	-	-
50-60%	0.7	50-60%	3.6
60-70%	0.7	60-70%	0.0
70-80%	1.6	70-80%	4.3
80-90%	2.9	80-90%	2.9
90-100%	18.2	90-100%	55.7
100%	75.6	100%	33.6

Supplementary Table 4. Correspondence of the plasma tau staging model using biomarker MS-determined ratios (p-tau/non-p-tau) vs biomarker concentrations (fmol/ml).

BioFINDER-2: 0.73				
	Stage 0 conc	Stage 1 conc	Stage 2 conc	Stage 3 conc
Stage 0 MS ratio	218	45	0	0
Stage 1 MS ratio	10	104	22	0
Stage 2 MS ratio	4	40	49	2
Stage 3 MS ratio	0	0	27	28
TRIAD: 0.69				
	Stage 0 conc	Stage 1 conc	Stage 2 conc	Stage 3 conc
Stage 0 MS ratio	37	3	0	0
Stage 1 MS ratio	6	25	8	1
Stage 2 MS ratio	1	12	17	0
Stage 3 MS ratio	0	0	12	18

Supplementary Table 5. Quantitative cross-stage profiles (median, IQR) for plasma p-tau217r, p-tau205r and 0N in BioFINDER and TRIAD.

Plasma stages	***0***	***1***	***2***	***3***	***All***
			BioFINDER		
p-tau217r	0.52 [0.39, 0.69]	0.81 [0.67, 1.21]	2.72 [2.28, 3.19]	3.66 [2.89, 4.7]	0.77 [0.52, 2.08]
p-tau205r	0.014 [0.011, 0.017]	0.021 [0.017, 0.025]	0.029 [0.025, 0.033]	0.035 [0.029, 0.04]	0.018 [0.014, 0.026]
Tau-0N	1.99 [1.46, 2.65]	3.98 [3.31, 4.93]	3.66 [2.68, 4.58]	7.60 [6.08, 8.95]	2.86 [1.96, 4.32]
			TRIAD		
p-tau217r	0.71 [0.48, 0.9]	1.88 [1.47, 2.12]	3.12 [2.87, 3.81]	5.06 [4.96, 5.25]	1.36 [0.77, 2.42]
p-tau205r	0.004 [0.003, 0.005]	0.007 [0.006, 0.009]	0.01 [0.009, 0.013]	0.017 [0.013, 0.017]	0.006 [0.004, 0.009]
Tau-0N	3.56 [2.57, 5.01]	5.79 [4.29, 8.17]	8.7 [7.06, 12.62]	14.85 [13.83, 16.11]	5.21 [3.31, 8.4]

Supplementary Table 6. Differences in AD biomarkers by plasma stages in the BioFINDER-2 and TRIAD cohorts.

	Stage 0 vs. 1		Stage 1 vs. 2		Stage 2 vs. 3	
	Standard β	p-value	Standard β	p-value	Standard β	p-value
**BioFINDER**						
**Aβ-PET**	0.37 [0.14. 0.59]	<0.001	1.34 [1.03. 1.65]	<0.001	-0.04 [-0.59. 0.5]	0.997
**Tau-PET early region (MTL)**	0.15 [-0.06. 0.36]	0.248	1.30 [1.04. 1.56]	<0.001	0.27 [-0.08. 0.62]	0.185
**Tau-PET intermediate region (NeoT)**	0.1 [-0.12. 0.32]	0.633	1.13 [0.87. 1.4]	<0.001	0.99 [0.63. 1.35]	<0.001
**Cortical thickness**	-0.27 [-0.52. -0.02]	0.027	-0.84 [-1.15. -0.53]	<0.001	-0.33 [-0.73. 0.07]	0.152
**mPACC**	-0.12 [-0.36. 0.11]	0.532	-0.92 [-1.24. -0.61]	<0.001	-0.55 [-0.99. -0.11]	0.008
**MMSE**	-0.08 [-0.31. 0.14]	0.78	-1.01 [-1.29. -0.73]	<0.001	-0.57 [-0.93. -0.21]	<0.001
**TRIAD**						
**Aβ-PET**	0.63 [0.25. 1]	<0.001	1.11 [0.72. 1.51]	<0.001	0.05 [-0.37. 0.47]	0.99
**Tau-PET early region (MTL)**	0.39 [-0.06. 0.83]	0.112	0.71 [0.25. 1.18]	0.001	0.47 [-0.03. 0.97]	0.073
**Tau-PET intermediate region (NeoT)**	0.14 [-0.33. 0.61]	0.868	0.73 [0.24. 1.23]	0.001	0.69 [0.15. 1.22]	0.006
**Cortical thickness**	-0.46 [-0.98. 0.07]	0.112	-0.51 [-1.07. 0.04]	0.081	-0.66 [-1.24. -0.07]	0.022
**CDR-SB**	0.30 [-0.22. 0.82]	0.446	0.48 [-0.07. 1.03]	0.115	0.49 [-0.1. 1.08]	0.142
**MMSE**	-0.26 [-0.8. 0.28]	0.6	-0.43 [-1. 0.14]	0.209	-0.48 [-1.09. 0.14]	0.186

Supplementary Table 7. Differences in slope of AD biomarkers by plasma stages in BioFINDER-2.

	Stage 0 vs. 1		Stage 1 vs. 2		Stage 2 vs. 3	
	Standard β	p-value	Standard β	p-value	Standard β	p-value
**Aβ-PET**	0.06 [0.04, 0.09]	<0.001	0.04 [0, 0.07]	0.058	0.01 [-0.06, 0.08]	0.747
**Tau-PET early region (MTL)**	0.02 [0, 0.05]	0.062	0.11 [0.08, 0.15]	<0.001	0.06 [0.01, 0.11]	0.031
**Tau-PET intermediate region (NeoT)**	0.02 [-0.01, 0.05]	0.122	0.23 [0.19, 0.27]	<0.001	0.13 [0.08, 0.19]	<0.001
**Cortical thickness**	-0.05 [-0.09, -0.01]	0.02	-0.19 [-0.24, -0.13]	<0.001	-0.17 [-0.25, -0.09]	<0.001
**mPACC**	-0.11 [-0.18, -0.03]	0.009	-0.42 [-0.53, -0.32]	<0.001	-0.19 [-0.34, -0.04]	0.014
**MMSE**	-0.08 [-0.15, 0]	0.054	-0.50 [-0.60, -0.41]	<0.001	-0.12 [-0.25, 0.01]	0.06

Supplementary Table 8. Tryptic tau peptides targeted in the study.

Respective peptide sequence, dominant charge state and monoisotopic m/z value.

Peptide	Peptide aa positions	Target peptide sequence	Charge state	*m/z*
P-tau181	175-190	TPPAPK[pT]PPSSGEPPK	3	556.606
Tau195-209	195-209	SGYSSPGSPGTPGSR	2	697.321
P-tau199	195-209	SGYS[pS]PGSPGTPGSR	2	737.304
P-tau202	195-209	SGYSSPG[pS]PGTPGSR	2	737.304
P-tau205	195-209	SGYSSPGSPG[pT]PGSR	2	737.304
Tau212-221	212-221	TPSLPTPPTR	2	533.798
P-tau217	212-221	TPSLP[pT]PPTR	2	573.781
P-tau231	225-240	KVAVVR[pT]PPKSPSSAK	3	577.989
0N-tau	045-126	AEEAGIGDTPSLEDEAAGHVTQAR	3	808.714
1N-tau	068-126	STPTAEAEEAGIGDTPSLEDEAAGHVTQAR	3	1004.134
PNS 131-138	131-138	VVQEGFLR	2	474.269
PNS 275-291	275-291	VSTEIPASEPDGPSVGR	2	849.421
