# Supplementary material for: Plasma tau biomarkers for biological staging of Alzheimer’s disease
Source: Nat Aging. 2025 Aug 22;5(11):2297–308. doi: 10.1038/s43587-025-00951-w (PMC12618263; doi:10.1038/s43587-025-00951-w)
Supplement: Supplementary file 2 — Reporting Summary [file 43587_2025_951_MOESM2_ESM.pdf]

nature portfolio

Corresponding author(s): Laia Montoliu-Gaya
Oskar Hansson

Last updated by author(s): Jul 18, 2025

Reporting Summary

Nature Portfolio wishes to improve the reproducibility of the work that we publish. This form provides structure for consistency and transparency in reporting. For further information on Nature Portfolio policies, see our Editorial Policies and the Editorial Policy Checklist.

Statistics

For all statistical analyses, confirm that the following items are present in the figure legend, table legend, main text, or Methods section.

n/a	Confirmed	
☐	☒	The exact sample size (*n*) for each experimental group/condition, given as a discrete number and unit of measurement
☐	☒	A statement on whether measurements were taken from distinct samples or whether the same sample was measured repeatedly
☐	☒	The statistical test(s) used AND whether they are one- or two-sided<br>*Only common tests should be described solely by name; describe more complex techniques in the Methods section.*
☐	☒	A description of all covariates tested
☐	☒	A description of any assumptions or corrections, such as tests of normality and adjustment for multiple comparisons
☐	☒	A full description of the statistical parameters including central tendency (e.g. means) or other basic estimates (e.g. regression coefficient) AND variation (e.g. standard deviation) or associated estimates of uncertainty (e.g. confidence intervals)
☐	☒	For null hypothesis testing, the test statistic (e.g. *F*, *t*, *r*) with confidence intervals, effect sizes, degrees of freedom and *P* value noted<br>*Give P values as exact values whenever suitable.*
☒	☐	For Bayesian analysis, information on the choice of priors and Markov chain Monte Carlo settings
☒	☐	For hierarchical and complex designs, identification of the appropriate level for tests and full reporting of outcomes
☐	☒	Estimates of effect sizes (e.g. Cohen's *d*, Pearson's *r*), indicating how they were calculated

Our web collection on statistics for biologists contains articles on many of the points above.

Software and code

Policy information about availability of computer code

Data collection	Thermo Scientific Xcalibur 4.5, Thermo Scientific Tune 3.5
Data analysis	Skyline 22.2, R (version 3.6.3) and Rminc (Version 1.5.2.3) software and their publicly available packages.

For manuscripts utilizing custom algorithms or software that are central to the research but not yet described in published literature, software must be made available to editors and reviewers. We strongly encourage code deposition in a community repository (e.g. GitHub). See the Nature Portfolio guidelines for submitting code & software for further information.

Data

Policy information about availability of data

All manuscripts must include a data availability statement. This statement should provide the following information, where applicable:

- Accession codes, unique identifiers, or web links for publicly available datasets
- A description of any restrictions on data availability
- For clinical datasets or third party data, please ensure that the statement adheres to our policy

This study includes no data deposited in external repositories. Anonymized data can be shared upon reasonable request from a qualified academic investigator, for the sole purpose of replicating procedures and results presented in the article, as long as data transfer agrees with local legislation and with the local Ethical Review Board of each cohort, which must be regulated in a material/data transfer agreement.

Research involving human participants, their data, or biological material

Policy information about studies with human participants or human data. See also policy information about sex, gender (identity/presentation), and sexual orientation and race, ethnicity and racism.

Reporting on sex and gender	sex was balanced between groups and was it accounted for in the relevant statistical analyses.
Reporting on race, ethnicity, or other socially relevant groupings	Race and ethnicity were not identified in this study because of the policy in BioFINDER-2 study.
Population characteristics	Population characteristics are described in Table 1.
Recruitment	BioFINDER-2 cohort included cognitively unimpaired participants (recruited as cognitively normal controls or as subjective cognitive decline [SCD] patients), patients with MCI, AD dementia patients and patients with a non-AD neurodegenerative disease. Participants were recruited at Skåne University Hospital and the Hospital of Ängelholm in Sweden. Details on recruitment, exclusion and inclusion criteria have been presented before.
Ethics oversight	All participants in the Swedish BioFINDER-2 cohort gave written informed consent and ethical approvals were granted by the Regional Ethical Committee in Lund, Sweden.

Note that full information on the approval of the study protocol must also be provided in the manuscript.

Field-specific reporting

Please select the one below that is the best fit for your research. If you are not sure, read the appropriate sections before making your selection.

☒ Life sciences ☐ Behavioural & social sciences ☐ Ecological, evolutionary & environmental sciences

For a reference copy of the document with all sections, see nature.com/documents/nr-reporting-summary-flat.pdf

Life sciences study design

All studies must disclose on these points even when the disclosure is negative.

Sample size	Samples were chosen based on the availability of fluid volume for analysis, age and clinical diagnosis availability.
Data exclusions	No data were excluded from the analyses.
Replication	Replication was done by repeating the reported analysis in 2 different cohorts. All attempts at replication were successful.
Randomization	Participants in the study were allocated in the different groups based on clinical diagnosis. Blood samples were randomized before preparation.
Blinding	Fluid analysis was done blind to any information regarding to clinical or imaging status.

Reporting for specific materials, systems and methods

We require information from authors about some types of materials, experimental systems and methods used in many studies. Here, indicate whether each material, system or method listed is relevant to your study. If you are not sure if a list item applies to your research, read the appropriate section before selecting a response.

Materials & experimental systems

n/a	Involved in the study	
☐	☒	Antibodies
☒	☐	Eukaryotic cell lines
☒	☐	Palaeontology and archaeology
☒	☐	Animals and other organisms
☒	☐	Clinical data
☒	☐	Dual use research of concern
☒	☐	Plants

Methods

n/a	Involved in the study	
☒	☐	ChIP-seq
☒	☐	Flow cytometry
☒	☐	MRI-based neuroimaging

Antibodies

Antibodies used	Tau12 (Purified anti-Tau, 6-18 Antibody, 806501, Biolegend)/ HT7 (Tau Monoclonal Antibody, MN1000, Thermofisher)/ BT2 (Tau Monoclonal Antibody, MN1010, Thermofisher
Validation	Tau12 (https://www.biolegend.com/en-us/search-results/purified-anti-tau-6-18-antibody-11569), HT7 (https://www.thermofisher.com/antibody/product/Tau-Antibody-clone-HT7-Monoclonal/MN1000), BT2 (https://www.thermofisher.com/antibody/product/Tau-Antibody-clone-BT2-Monoclonal/MN1010)

Plants

Seed stocks	*Report on the source of all seed stocks or other plant material used. If applicable, state the seed stock centre and catalogue number. If plant specimens were collected from the field, describe the collection location, date and sampling procedures.*
Novel plant genotypes	*Describe the methods by which all novel plant genotypes were produced. This includes those generated by transgenic approaches, gene editing, chemical/radiation-based mutagenesis and hybridization. For transgenic lines, describe the transformation method, the number of independent lines analyzed and the generation upon which experiments were performed. For gene-edited lines, describe the editor used, the endogenous sequence targeted for editing, the targeting guide RNA sequence (if applicable) and how the editor was applied.*
Authentication	*Describe any authentication procedures for each seed stock used or novel genotype generated. Describe any experiments used to assess the effect of a mutation and, where applicable, how potential secondary effects (e.g. second site T-DNA insertions, mosiacism, off-target gene editing) were examined.*
